# Quantitative model for inferring dynamic regulation of the tumour suppressor gene p53

**DOI:** 10.1186/1471-2105-11-36

**Published:** 2010-01-19

**Authors:** Junbai Wang, Tianhai Tian

**Affiliations:** 1 Division of Pathology, The Norwegian Radium Hospital, Rikshospitalet University Hospital, Montebello 0310 Oslo, Norway; 2 School of Mathematical Sciences, Monash University, Melbourne, Vic 3800, Australia; 3 Department of Mathematics, University of Glasgow, Glasgow G12 8QW, UK

## Abstract

**Background:**

The availability of various "omics" datasets creates a prospect of performing the study of genome-wide genetic regulatory networks. However, one of the major challenges of using mathematical models to infer genetic regulation from microarray datasets is the lack of information for protein concentrations and activities. Most of the previous researches were based on an assumption that the mRNA levels of a gene are consistent with its protein activities, though it is not always the case. Therefore, a more sophisticated modelling framework together with the corresponding inference methods is needed to accurately estimate genetic regulation from "omics" datasets.

**Results:**

This work developed a novel approach, which is based on a nonlinear mathematical model, to infer genetic regulation from microarray gene expression data. By using the p53 network as a test system, we used the nonlinear model to estimate the activities of transcription factor (TF) p53 from the expression levels of its target genes, and to identify the activation/inhibition status of p53 to its target genes. The predicted top 317 putative p53 target genes were supported by DNA sequence analysis. A comparison between our prediction and the other published predictions of p53 targets suggests that most of putative p53 targets may share a common depleted or enriched sequence signal on their upstream non-coding region.

**Conclusions:**

The proposed quantitative model can not only be used to infer the regulatory relationship between TF and its down-stream genes, but also be applied to estimate the protein activities of TF from the expression levels of its target genes.

## Background

Transcription of genes is generally controlled by a regulatory region of DNA located mostly up-stream of the gene transcription start site. This regulatory region contains a short sequence that the regulatory proteins bind to in order to enhance/inhibit the gene expression [1]. Current advance in high-throughput technologies such as DNA microarrays, together with the availability of whole genome sequence for several species, enable us to study the genome-wide genetic regulatory networks. These heterogeneous functional genomic datasets have been used to acquire, catalogue and infer genetic regulatory networks in a "top-down" fashion. It focuses on the reverse-engineering of genetic networks by identifying the regulatory interactions, inferring the transcriptional modules and predicting the combinatorial regulation of transcriptional factors (TFs) [2-5]. On the contrary, another principal research method, namely the "bottom-up" approach, builds detailed mathematical models for small-scaled genetic regulatory networks based on extensive experimental observations. To accomplish that goal, various types of models have been proposed to describe the genetic regulation. These models include, for example, differential equation models with continuous-time and continuous-variables, Bayesian network models with discrete-time and continuous-variables and Boolean network models with discrete-time and discrete-variables. Particularly, many differential equation models (e.g. linear systems, neural networks, S-systems and nonlinear models) have been used to investigate the dynamic properties of genetic regulation [6-9].

One of the major challenges of using a "bottom-up" approach to infer genetic regulation from microarray datasets is the lack of information for protein concentrations and activities. Most of the previous researches were based on the assumption that the expression levels of a gene are consistent with its protein activities, though we know that is not always the case. An earlier practice to rectify above assumption is a hidden variable dynamic modelling (HVDM) method, which is a linear dynamic model designed to estimate the activities of a TF by using the expression activities of its target genes [10]. Later, the HVDM method was extended to a nonlinear one by using the Michaelis-Menten function [11]. In addition, mathematical models with time delay were also used to elucidate the time difference between the activities of TFs and the expression profiles of target genes [12,13]. Nevertheless, a more sophisticated inference method, which considers both the time delay and protein-DNA binding structure, is needed to accurately describe the genetic regulation in a "bottom-up" fashion. The development of such methods still remains as one of the major challenges in the computational study of genetic regulatory networks by the integration of "omics" datasets and experimental results [14,15].

In earlier works, several "bottom-up" researches used the "master" gene networks to validate their proposed inference methodologies, as well as to investigate the regulatory function of the "master" gene [9,10]. Among them, tumour suppressor gene p53 has been described as "the guardian of the genome" highlighting its role in conserving stability by preventing genome mutation. Since a point mutation within the p53 gene occurs in over half of all human tumours, an elucidation of the regulatory mechanisms of p53 gene will contribute tremendously to the development of strategies for treating cancer [16]. Although many experimental methods have been employed to identify the transcriptional target genes of p53 (e.g. the clustering analysis of microarray data [17], protein expression profiles [18] and Chip-PET identification of transcriptional-factor binding sites [19]), it is imperative to develop more sophisticated mathematical models that precisely describe the p53 regulation. In this work, we propose a nonlinear differential equation model, which considers both the protein-DNA binding structure and the effect of time delay, to infer genetic regulation from microarray gene expression datasets. The proposed method is then applied to predict the p53 target genes.

## Results

### Microarray data analysis

#### Preprocessing of raw microarray data

By using a previously published dataset [10], we selected 1,312 probes (e.g. the top 15% of the most responsive to the p53 activation, [Additional file 1]) from the preprocessed microarray dataset (~8,737 probes) by using the pair-wise Fisher's linear discriminant method [20]. To assess the robustness of such selection, we compared the gene selections between the pair-wise Fisher's linear discriminant method and the maSigPro method [21]. The maSigPro method is an R package especially designed for analyzing time-course microarray experiments, which was applied to the same preprocessed microarray dataset. The parameter settings of the maSigPro method are a false discovery value (Q) that equals to 0.05 and an R-squared threshold (R) whose value ranges from 0.3 to 0.9. Table 1 suggests that both methods converged when a higher R-squared threshold (e.g. *R *> 0.5 represents a good model fitting in the original paper of the maSigPro method [21]) is used. Particularly, with a higher R-squared threshold, genes provided by the maSigPro method overlap more (> 85%) with that selected by the Fisher's method. Thus, the defined top 15% of the most relevant response probes is considered to be a robust selection.

**Table 1:** A comparison of significantly differential gene selections between the pair-wise Fisher's linear discriminant method and maSigPro method.

(Q, R)	Genes selected by maSigPro	Genes overlap with our selection	Percent of overlapping
(0.05, 0.3)	1165	646	0.55

(0.05, 0.4)	1084	616	0.57

(0.05, 0.5)	661	455	0.69

(0.05, 0.6)	306	263	0.86

(0.05, 0.7)	139	131	0.94

(0.05, 0.8)	43	40	0.93

(0.05, 0.9)	14	12	0.86

#### Clustering analysis

Consequently, the selected 1,312 probes were assigned to 40 co-expressed gene modules by using a published computational approach [5,20] that combines the stress function, neuron gas algorithm and K-nearest neighbour method. Each gene module represents a set of co-expressed genes that are stimulated by either a specific experimental condition or a common trans-regulatory input. From a functional analysis of the 40 gene modules, we found that the co-expressed gene modules might contain genes with either heterogeneous or homogeneous biological functions, which are irrelevant to the number of genes in each module. Rather, it may reflect the complex mechanisms that control the transcription regulation. Therefore, in the subsequent analysis, we applied our nonlinear dynamic model on the profile of each individual gene instead of the mean centre of each gene module. Detailed information of 1,312 probes and the corresponding 40 co-expressed gene modules are available in [Additional file 1].

### Validation of mathematical model

#### Predicting protein activity from microarray gene expression profiles

**Figure 1:**
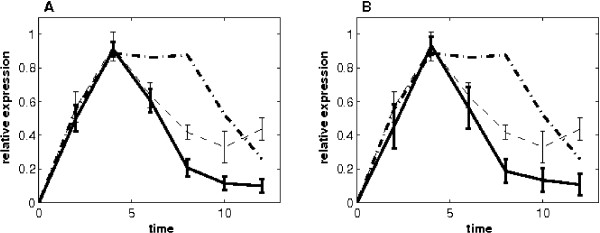
**Estimated p53 activity and the 95% confidence intervals based on five training genes (DDB2, PA26, TNFRSF10b, p21 and Bik) that are positively regulated by P53**. (A) Estimates from the three replicates of microarray expression data. (B) Estimates from the mean of the three-replicate expression data. (Dash-dot line: p53 activities measured by Western blot [10]. The protein level p53 activation come a time-course immunoblot examination of p53 phosphorylated on S15; dash line: estimate of the HVDM method; solid line: prediction of the nonlinear model.)

Based on the p53 protein-DNA binding structure, we developed a nonlinear dynamic model (5) with a Hill function to represent the expression process of p53 target genes. The Hill coefficient was chosen to be 4 because p53 is in the form of tetramer as a transcription factor [22]. In addition, the proposed nonlinear model enables us to infer target genes that are negatively regulated by p53. In an earlier work, a linear model provided good estimation of p53 activities by using five known p53 target genes [10]. To evaluate the performance of our nonlinear model, we used the same p53 targets (i.e. DDB2, PA26, TNFRSF10b, p21 and Bik which are all positively regulated by p53) to predict the activities of p53. Here the time delay was assumed to be zero due to performing a consistent comparison study between the two models. Ten sets of unknown model parameters together with the p53 activities at 6 time points were estimated from each replicate of the 3 microarray experiments and also from the average of these 3 microarray time courses. Figure 1A presents the mean and 95% confidence interval of the 30 sets of the predicted p53 activities from 3 microarray experiments, and Figure 1B shows the results of the 10 predictions from the averaged time courses of 3 microarray experiments. The relative error of the estimate in Figure 1B is 2.70, which is slightly larger than both that in Figure 1A (2.70) and that obtained by the linear model (1.89). From Figure 1, we found that the new nonlinear model achieves the same goal as the linear model for predicting p53 activities.

#### Accessing the predicted protein activity from various training genes

**Figure 2:**
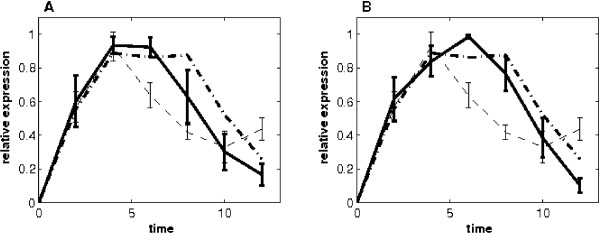
**Estimated p53 activity and the 95% confidence intervals based on five training genes (RAD21, CDKN3, PTTG1, MKI67 and IFITM1) that are negatively regulated by P53**. (A) Estimates from the three replicates of microarray expression data. (B) Estimates from the mean of the three-replicate expression data. (Dash-dot line: p53 activities measured by Western blot [10]; dash line: estimate of the HVDM method; solid line: prediction of the nonlinear model.)

To determine the influence of training genes on the estimation of p53 activities, we selected various sets of 5 training genes to infer the p53 activities. Although the obtained p53 activities in one test are similar to those presented in Figure 1, in which 5 training genes [23] are negatively regulated by p53, there is slight difference between the estimated p53 activities by using different sets of training genes. One of the tests is shown in Figure 2, where the estimated p53 activities were based on 5 training genes (RAD21, CDKN3, PTTG1, MKI67 and IFITM1) that are negatively regulated by p53 [24-26]. Similar to the study presented in Figure 1, ten sets of the p53 activities were estimated from each replicate of the 3 microarray experiments and also from the average of these 3 microarray time courses. The mean and 95% confidence interval of both estimates are presented in Figures 2A and 2B, respectively. The relative error of the estimate in Figure 2B is 1.28, which is very close to that in Figure 2A (1.30) but smaller than that obtained by the linear model (1.89) in Figure 1. In this case, the estimated p53 activities are very close to the measured ones. It suggests that our proposed nonlinear model is capable of making reliable predictions for the TF activities from the training genes that are all either positively or negatively regulated by the TF p53, though the dependence between the training genes and predicted TFs activities may exist.

#### Sensitivity analysis of model parameters

**Figure 3:**
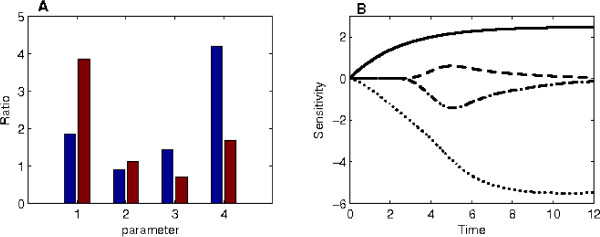
**Sensitivity analysis of the nonlinear mathematical model**. (A) Ratios of the simulation errors obtained by varying one of the model parameters (index 1: parameter *c*; 2: *k*; 3: *K*; 4: *d*. Left bar: the parameter is decreased (*k *= 0.9*k*); right bar: the parameter is increased (*k *= 1.1*k*)). (B) The drift of the solution with a unit perturbation of one model parameter obtained by using the Khalil method (solid-line: parameter *a*; dash-line: *k*; dash-dot line: *K*; dot-line: *d*).

For the proposed nonlinear model (5), we also tested the variation of system dynamics by changing one of the four reaction rates (*c*_*i*_, *k*_*i*_, *K*_*i*_, *d*_*i*_). In this test, we used the predicted p53 activities and the corresponding model parameters to simulate the expression levels of gene DDB2 in Figure 3A. By tuning one of the four parameters (e.g. either increasing or decreasing its value by 10%), we measured the ratio of simulation errors, defined by(1)

where *x*(12) is the microarray expression level at *t *= 12, *x*_12 _is the simulated expression level from the estimated model parameter, and  is the simulated expression level from the perturbed model parameter. Figure 3A indicates that an increasing of the basal expression rate *c*_*i *_and a decreasing of the degradation rate *d*_*i *_will cause considerable changes in the simulations (e.g. error ratios 3.84 and 4.19, respectively.) In addition, modification of maximal expression rate *k*_*i *_induces similar changes in the simulation; and a decreasing in parameter *K*_*i *_causes an error ratio 1.44. Furthermore, we used the Khalil method [27] to investigate the influence of parameter variations on the system dynamics at the other time points. Simulations in Figure 3B represent the variation of system dynamics induced by a unit perturbation of model parameter, which is similar to the defined ratio (1). The results of the sensitivity analysis in Figure 3B are consistent with those in Figure 3A. Therefore, a variation of any parameter in the proposed nonlinear model may have considerable influence on the system dynamics.

### Prediction of p53 target genes

#### Effect of time delay in p53 target gene prediction

In order to make a new prediction rather than reproduce the published results, we used the newly inferred p53 activity in Figure 2B and the nonlinear model (5) to study the genetic regulation of p53 target genes. In the new model, the maximal possible time delay was set to 2.5 hours because the experimentally determined time delay for p53 target genes is up to 2 hours [17,28]. We used the genetic algorithm to infer the p53 mediated genetic regulation (see Methods for detailed information). In different implementations of the genetic algorithm, the additionally unknown parameter of time delay may cause the estimates to vary across a wide range of values. To reduce such parameter variation, we used a natural spline interpolation to expand the measurements from the original 7 time points to 25 time points, by adding three equidistant measurement points between each pair of measured time points. In addition, we estimated the genetic regulation twice for each gene (e.g. either with or without time delay), and selected a final regulation result which has the smallest model estimation error.

#### Comparison of predicted regulation states of p53 target genes across three different methods

Subsequently, both the event method [29] and correlation approach [5] were used to infer the activation/inhibition of the p53 regulation. By comparing the consistency of inferred regulation relationships among the three methods (i.e. the nonlinear model, event method and correlation method), we found that 657 and 423 of 1,312 probes from the estimation of the nonlinear model overlap with the results by the correlation method and event method, respectively. However, only 241 genes have the same p53 regulation state across all three methods. For the top 656 probes (50%) that with smaller model estimation errors, the number of overlapping probes among the three methods is reduced to 414, 265 and 166, respectively. If we reduced the probe number further by considering the top 328 genes (25%) only, then the overlapping number is reduced to 206, 130 and 80, respectively. Thus, by reducing the probe number from 1,312 to 656, the proportions of gene numbers with the same predicted p53 regulation was increased (e.g. 414:656 is greater than 657:1312). However, by reducing the probe number further to 328, we did not find such change. Therefore, in subsequent data analysis, we only focused on the top 656 (~50%) predicted genes. Among these putative p53 target genes, ~64% are positively regulated by p53 while the rest are negatively regulated. A GO functional study of these 656 putative p53 target genes indicates that ~16% of them have unknown functions and these genes are excluded from our further study.

#### Binding motif information of predicted p53 target genes

To provide more criteria for identifying putative p53 target genes, we searched for the p53 binding motif on the upstream non-coding region of the top 656 genes. This is because a physical interaction between p53 and its targets is essential for its role as a controller of the genetic regulation [1]. Particularly, p53 has a well documented 10 bp consensus binding motif (RRRCWWGYYY) and a DNA sequence with two copies of such monomer is strongly bound by the p53 protein [1]. Thus, for each putative target, we extracted the corresponding 10 kb DNA sequences located directly upstream of the transcription start site from Refs [30]. Among the 656 putative p53 target genes, we found the upstream DNA sequences for 511 of them. Then a motif discovery program MatrixREDUCE [31] was applied to search for the p53 consensus binding site. The results indicate that ~72.0% (366 out of 511 genes) of putative p53 targets have at least 2 copies of the p53 binding motif (perfect match counts of p53 binding site), while only ~10% (47 out of 511 genes) and ~20% (98 out of 511 genes) of them have zero and one p53 monomer, respectively. Based on the model estimation error and upstream TF-binding information of the 656 putative p53 target genes, we further narrowed down the number of possible p53 targets. In addition, for any gene that has more than one probe, we chose only the probe that has the smallest estimation error. We also excluded genes with very small parameter *k*_*i *_in model (5) because p53 may not have much influence on them [10]. A final list containing ~317 putative p53 targets [Additional file 2] covers around ~24% of the total studied probes (~1312). Table 2 presents 50 of these predicted putative p53 target genes.

**Table 2:** Putative p53 target genes predicted by our method.

	Order	Probe Set ID	Gene Symbol	error/regulation		Order	Probe Set ID	Gene Symbol	error/regulation
1	1	217732_S_AT	ITM2B	0.0382 (+)	26	87	202431_S_AT	MYC	0.6894 (-)
2	2	205347_S_AT	TMSL8	0.0828 (+)	27	91	203509_AT	SORL1	0.7450 (+)
3	3	211630_S_AT	GSS	0.1100 (+)	28	125	219863_AT	HERC5	1.0149 (+)
4	4	201202_AT	PCNA	0.1148 (+)	29	132	205692_S_AT	CD38	1.0689 (+)
5	5	208812_X_AT	HLA-C	0.1216 (+)	30	141	213204_AT	PARC	1.1296 (+)
6	6	202649_X_AT	RPS19	0.1396 (+)	31	145	209375_AT	XPC	1.1468 (+)
7	7	211714_X_AT	TUBB	0.1495 (+)	32	147	201834_AT	PRKAB1	1.1574 (+)
8	9	201761_AT	MTHFD2	0.1848 (-)	33	152	209849_S_AT	RAD51C	1.2028 (+)
9	10	202605_AT	GUSB	0.1933 (+)	34	159	219361_S_AT	ISG20L1	1.2623 (+)
10	11	209140_X_AT	HLA-B	0.1956 (+)	35	178	204958_AT	PLK3	1.4262 (-)
11	12	210968_S_AT	RTN4	0.1996 (-)	36	185	205266_AT	LIF	1.5033 (+)
12	13	201476_S_AT	RRM1	0.2046 (+)	37	202	202729_S_AT	LTBP1	1.6431 (+)
13	14	204026_S_AT	ZWINT	0.2087 (+)	38	203	213293_S_AT	TRIM22	1.6431 (+)
14	18	216705_S_AT	ADA	0.2235 (+)	39	205	204321_AT	NEO1	1.6711 (+)
15	20	202503_S_AT	KIAA0101	0.2318 (+)	40	215	205043_AT	CFTR	1.7426 (+)
16	21	218740_S_AT	CDK5RAP3	0.2382 (+)	41	234	213523_AT	CCNE1	1.9631 (+)
17	23	213060_S_AT	CHI3L2	0.2785 (+)	42	244	218611_AT	IER5	2.0564 (-)
18	24	221943_X_AT	RPL38	0.2858 (+)	43	257	202284_S_AT	CDKN1A	2.1920 (+)
19	25	218883_S_AT	MLF1IP	0.2891 (+)	44	275	208478_S_AT	BAX	2.3018 (+)
20	30	201721_S_AT	LAPTM5	0.3121 (+)	45	277	202095_S_AT	BIRC5	2.3172 (-)
21	31	208149_X_AT	DDX11	0.3408 (+)	46	279	204009_S_AT	KRAS	2.3263 (-)
22	32	209773_S_AT	RRM2	0.3454 (+)	47	290	203752_S_AT	JUND	2.4195 (+)
23	33	218403_AT	TRIAP1	0.3500 (+)	48	306	217373_X_AT	MDM2	2.5517 (-)
24	36	201577_AT	NME1	0.3645 (+)	49	309	211725_S_AT	BID	2.5805 (-)
25	38	210774_S_AT	NCOA4	0.3703 (+)	50	312	203725_AT	GADD45A	2.6107 (+)

The first 25 genes have minimal model error and the other 25 genes are known p53 target genes. (+: gene is activated by p53; -: gene is inhibited by p53)

#### Discrepancies between different predictions

It is interesting to explore whether the putative p53 target genes identified above correspond to sets that have been discovered by other methods. For that reason, we collected four lists of putative p53 targets from different studies. They are 45 unique genes from 50 predicted p53 target probes which were obtained by applying the linear HVDM method on the Affymetrix microarray time-series data [10]; 317 unique genes which were detected by applying our non-linear dynamic model on the above same dataset; 76 unique genes which were identified by analysing p53-regulated gene expression profiles of oligonucleotide arrays [17]; and 205 unique genes which were suggested by Chip-PET analysis of human genome-wide p53 transcription-factor binding sites [19]. As shown in Table 3, the overlapping among the different predictions is quite poor.

**Table 3:** The number of overlapping genes between the predicted putative p53 targets from the MVDM method [10], gene expression analysis (GRA) [17], Chip-PET analysis [19] and our nonlinear model in this work.

	MVDM	GRA	Chip-PET	Nonlinear
MVDM	45	4	14	27

GRA	4	76	13	10

Chip-PET	14	13	205	21

Nonlinear	27	10	21	317

#### Target gene bias from microarray datasets

To find out the reason for these discrepancies, we examined the 76 target genes that were identified in reference [17]. Among these 76 target genes, 31 of them were firstly removed in our pre-processing step due to the weak signals, bad quality or less variation across all time points. Secondly, another 31 of them were removed in the later selection of the most relevant response probes by using the Fisher's linear discriminant method because of their weak response to the ionizing radiation. In the end, only 14 of the 76 genes were entered into our nonlinear model and we finally identified 10 of them as our putative p53 target genes (e.g. CDKN1A, MST1 and BIRC5 in [Additional file 2]). The remaining 4 genes such as HSD17B1 were not included in our prediction because of the relatively large model estimation errors. The large errors may be a by-product of the noise in the microarray gene expression data.

#### Target gene bias from inference models

**Figure 4:**
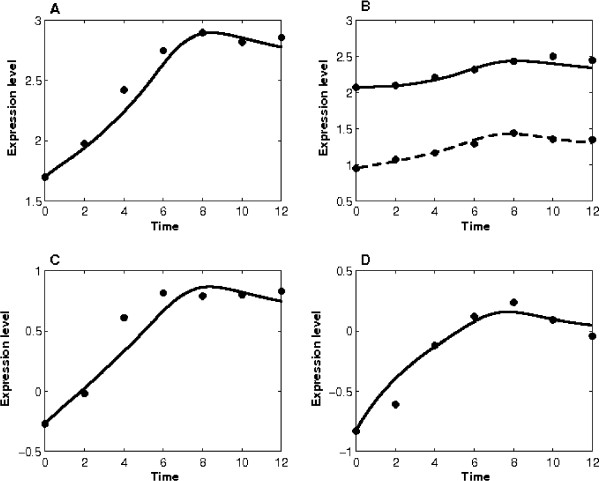
**Expression profiles of four p53 target genes that were identified by the HVDM method**. (A) Gene DENND2D (probe ID 221081_S_AT) was also predicted in this work. (B) Gene RRM1 has two probe set ID 201477_S_AT (dash-line, predicted by the HVDM method) and 201476_S_AT (solid-line, not listed in [10]). Probe 201476_S_AT has smaller model error and thus is listed in [Additional file 2]. Probe 201477_S_AT was removed from our prediction. (C) Gene CROT (probe ID 204573_AT) was removed from our consideration because it has no p53 binding motif on its regulatory region. (D) Gene GAL3ST4 (probe ID 219815_AT) was excluded from our consideration because it has relatively larger model estimation error. (Line: simulation of the nonlinear model, star: microarray gene expression profiles.)

We also investigated the 50 putative target genes that were provided by the linear model [10]. First of all, 48 of them were included in the top 15% of the most relevant response probes (~1,312 probes). Secondly, 36 of them were within the top 50% of the 1,312 probes and we removed 12 genes due to the relatively larger model estimation errors. Finally, we further discarded probes with duplicate gene names (~2) and genes without p53 binding site on the regulatory region (~7). Therefore in the final list in [Additional file 2], we presented only 27 of the remaining 36 genes. Figure 4 shows both the predicted and measured expression profiles of 4 genes which were selected in reference [10]. Taken together, we conclude that the discrepancy of p53 target gene predictions among various studies may be mainly caused by either pre-processing of microarray data or condition-specific gene regulation.

#### *In silico *validation of putative p53 targets

Although a wet lab experiment may be the best way to validate the whole list of predictions, other external information such as DNA sequence analysis could be used to support the computational predictions [32]. For example, we found ~80% of the top 317 putative p53 targets have at least 2 perfect matches of p53 consensus sequences (RRRCWWGYYY) on the 10 Kb upstream region. This may support the hypothesis that the predicted target genes may be strongly bound by p53 in vivo [1]. A short list of these p53 target genes is shown in Table 2, where we found many known p53 target genes including p21, Bax, Bik and Mdm2. However, a number of the top ranked putative target genes, such as the 4 genes (RPS19, RPL38, RPS27L, and RPL37A) that encode ribosomal proteins and several major histocompatibility complex genes (e.g. HLA-C and HLA-B), seem to have no obvious connection to p53. These ribosomal proteins have been shown to activate p53 by inhibiting oncoprotein MDM2, leading to inhibition of cell cycle progression [33]. Thus, the ribosomal proteins can regulate the p53-MDM2 feedback loop in response to different stresses and provide a general pathway for p53 activation from perturbation of ribosome biogenesis. For major histocompatibility complex genes, they are involved in the major histocompatibility complex (MHC) class I antigen presentation pathway which plays a key role in host tumour surveillance. Experimental data suggest that p53 activates the MHC class I pathway by inducing TAP1, which would assist the process [34].

### Protein binding motif analysis for putative p53 target genes

#### Binding site distribution of putative p53 target genes

The lack of common p53 targets among four different predictions generated a few interesting questions to us. Will the four lists of putative p53 targets share the same p53 binding motif distribution on the upstream non-coding region? Will the genes predicted from these four studies share the same functional categories too? To answer these questions, we collected the p53 binding motif counts on the gene upstream regions for the four predictions and listed the results in Table 4. It indicates that putative targets predicted by the gene expression analysis, the Chip-PET analysis, and our nonlinear model, share a similar p53 binding preference. For example, there is an even distribution (~20%) of zero, one, two, and more than two p53 binding sites on the 5 kb region. However, there are more p53 binding motifs on the 10 kb upstream region than those on the 5 kb region. In addition, ~46-58% of putative p53 targets have more than two p53 binding sites on the 10 kb upstream region but only ~16-20% of targets have multiple binding sites on the 5 kb region. Furthermore, less than 10% of targets do not have p53 binding sites on the 10 kb region. The similar binding preference among various predictions suggests that the majority of putative p53 targets (~70%) may be directly controlled by remote p53 transcription factors but less than 30% of them may be the second effect targets.

**Table 4:** Comparison of the p53 consensus motif distributions in the four sets of putative p53 target genes obtained by the MVDM method [10], gene expression analysis [17], Chip-PET analysis [19] and our nonlinear model in this work.

# of Perfect match	MVDM	GRA	Chip-PET	Nonlinear
0 p53 motif (5 k)	0.41	0.24	0.28	0.22

1 p53 motif (5 k)	0.22	0.38	0.32	0.33

2 p53 motif (5 k)	0.24	0.20	0.25	0.23

> 2 p53 motif (5 k)	0.14	0.18	0.16	0.20

0 p53 motif (10 k)	0.25	0.08	0.06	0.05

1 p53 motif (10 k)	0.14	0.19	0.24	0.15

2 p53 motif (10 k)	0.20	0.27	0.23	0.22

> 2 p53 motif (10 k)	0.41	0.46	0.47	0.58

#### Functional analysis of putative p53 target genes

A functional analysis of above four lists of putative p53 targets also reveals interesting information such as the fact that all works identified the same core biological functions of p53 (e.g. cell cycle, cell death, cell proliferation and response to DNA damage stimulus). However, there are a few gene functional categories that were only predicted by individual studies. For example, the lists from the gene expression analysis and Chip-PET analysis contain blood coagulation, body fluids, response to wound, muscle and signal transduction genes. However, only the list from the Chip-PET analysis is enriched by cell motility, cell localization and enzyme activity genes. In addition, high enrichment of metabolism, biosynthetic process and immune system process exclusively appear in our prediction. Although our results indicate that most of the p53 targets share the same p53 binding preference, their functional roles are conditionally specific and their biological functions span to various functional categories with the dependence of intrinsic and extrinsic conditions. The functional differences among the four lists of putative p53 targets may partially explain the reason for the poor overlapping among them. In addition, the poor overlapping may be caused by the putative p53 target genes that were induced by different types of event such as different cell types or different treatments of p53 regulation. For example, the target genes identified by microarray time-series data [10] was under γ-irradiated Human MOLT4 cells; but the target genes predicted from oligonucleotide arrays [17] and ChIP-PET analysis [19] were induced by zinc-induced p53 in EB-1 cells and 5-fluorouracil treated HCT116 cells, respectively. Thus, the results suggest that the nature of p53 response is conditionally dependent. Different experiments form distinct sets of putative target genes and a subset with a few target genes in common to all p53 responses [17].

#### Combinational regulation of putative p53 target genes

Furthermore, we looked for the potential p53 co-regulators on the upstream non-coding region of the putative p53 target genes. By collecting 409 weight matrixes of human transcription-factors, which represent 254 unique human TFs from the TRANSFAC database [35], and transforming the weight matrices to the position-specific affinity matrices, we used the MatrixREDUCE program [31] to compute the transcription factor binding affnities on the upstream of all putative p53 targets [Additional file 3]. A clustering analysis of the relative sequence affinity profiles for human TFs was also performed [Additional file 4], which suggests the predicted sequence signals of several human TFs are either commonly enriched or depleted in all targets related to the expected occurrence on random sequences. For instance, the top two most depleted sequence signals are E2F and CREB, which rarely appear on the 10 k upstream region of all putative p53 target genes. It suggests that these two TFs may not directly interact with p53 target genes. Such hypothesis is consistent with the literature information, which claims that both E2F and CREB often interact with other proteins directly and form a protein complex to regulate the transcriptional activity (e.g. the E2F-p53 complex stimulates the apoptotic function of p53 [36] and CREB modulates p53 by acetylation [37]). On the other hand, the top three most enriched sequence affnities on 10 kb upstream region for putative p53 targets are PITX2, FOXO1 and TBP, which are all known to be related to functional regulation of p53. PITX2 can bind to HPV E6 protein and inhibit E6/E6AP-mediated p53 degradation [38]. FOXO1 may function as a tumour suppressor and regulators of FOXO1 function are controlled by p53 [39]. TBP is a TATA-binding protein but p53 can prevent TBP from participating in RNA pol III-dependent transcription [40]. Thus, p53 response genes may preserve certain sequence specific features (e.g. a common *cis*-regulatory module on the upstream region) that enables p53 to interact easily with other co-regulators to control diverse biological processes.

## Discussion

This work developed a nonlinear model for inferring genetic regulation from microarray gene expression data. The major feature of this approach is the inclusion of the cooperative binding of TFs by which we can study the nonlinear properties of gene expression in a sophisticated way. It is also a practical approach to investigate the impact of time delay of gene expression on the dynamics of the down-stream target genes. We validated the proposed method by comparing the estimated TF p53 activities with experimental data. In addition, the predicted putative p53 target genes by our nonlinear model were supported by DNA sequence analysis which suggests that p53 predominately controls remote genes. The long-distance gene regulation may be accomplished by a cooperative regulation between p53 and other proteins. This hypothesis may also explain the poor overlap among the four lists of the putative p53 target genes, and support the fact that we could not find a p53 binding motif on the upstream non-coding region of at least 20% of the putative p53 targets although these genes may be strongly positively regulated by p53 protein [10].

For issues regarding the estimation of protein activities and the effects of time delay in genetic regulation, we first emphasize that gene transcription depends on multiple factors such as the activities of transcription factors, the availability of RNA polymerase and the activities of other promoters in the transcriptional machinery. For example, in order to activate gene expression, the required availability of RNAP II and other promoters differ significantly between two p53 target genes - p21 and Fas/AOP1 [28]. However, most modelling approaches including our current study approximate the activities of all the promoters in the transcriptional machinery as the activities of TF p53. Therefore, our estimated p53 activities represent the total activities of all factors in the transcriptional machinery, which may be slightly different from one another if various sets of training target genes were used. In addition, time delay exists in many biological processes of gene expression such as transcriptional initiation, elongation, protein translocation, and translational elongation. However, in the present model, we simplified all kinds of time delay effects into a single factor. This is a practical approach to study the time delay effect of each individual p53 target gene, and therefore the time delay of each gene may differ.

Finally, a number of factors may contribute to the variation of predictions using the mathematical model. For example, we have shown that the selection of training genes may influence the estimation of p53 activity and consequently alter the prediction of putative target genes. In addition, the proposed nonlinear differential equation model may affect the estimation of putative target genes. The present model estimation error is related to the selection of synthesis function. Although the Michaelis-Menten function is generally used if there is no extra information about the TFs, more precise estimates may be obtained by using a more sophisticated synthesis function which requires TFs' cooperative binding and/or binding sites information. Furthermore, in the present work the relative error was used to compare the errors of different genes. Nevertheless, the model estimation error may be large if the gene expression is weak. For that reason, a number of discovered p53 target genes were not included in our prediction, even though their simulations matched well the gene expression profiles (Figure 4D). Thus, it is worthy to evaluate the influence of the error measurement on both the predictions of the TF activities and genetic regulation to the putative target genes. In that case, other error measurement methods (e.g. the weighted distance measure [41]) may be considered. Finally, it is widely recognized that microarray gene expression data is noisy. It is therefore important to develop stochastic models and the corresponding stochastic inference methods [42] to investigate the impact of gene expression noise on the accuracy of the modelling inference.

## Conclusions

In summary, we have developed a nonlinear model for inferring genetic regulation from microarray gene expression data. This "bottom-up" method was designed not only to infer the regulation relationship between TF and its down-stream genes but also to estimate the up-stream protein activities based on the expression levels of the target genes. The successful prediction of a large number of putative p53 target genes indicates that the proposed dynamic model is a promising method to investigate genetic regulation. It is expected that our results will provide both valuable prediction for further experimental validation and quantitative information for the development of the p53 gene regulatory networks.

## Methods

### Microarray data analysis

This research is based on a published microarray dataset which was generated from the Human All Origin, MOLT4 cells carrying wild-type p53. Cell were *γ*-irradiated and harvested every 2 hours over a 12-hours period [10]. We obtained the ionizing radiation Affymetrix dataset [10] from ArrayExpress (E-MEXP-549). Firstly, the microarray dataset was pre-processed by an R BioConduct package [43], in which probes with bad signal quality and less variation across all the time points were removed. This resulted in ~8,737 probes from a total of 22,284 probes. The pre-processed probes were then further median centred within each array and transformed to Z-scores before using the pair-wise Fisher's linear discriminant method [20] to screen probes with the most relevant response to ionizing radiation. The top 15% of the most relevant response probes (~1,312 probes) were selected as the input data to our nonlinear model. All gene symbols were obtained from the NETAFFX [44]. It is noteworthy that 2 of 50 putative p53 target probes (201714_at and 220623_s_at) from Refs [10] are not included in the selected 1,312 probes.

### Nonlinear model

A mathematical model with a general type of the *cis*-regulatory functions has been proposed recently aimed at reconstructing genetic regulatory networks [12,13]. The model includes both positive and negative regulation, time delay and number of DNA-binding sites. However, the cooperative binding of TFs was not considered. In this work, we propose a new model where the dynamics of gene transcription is represented as(2)

where *c*_*i *_is the basal transcriptional rate, *k*_*i *_is the maximal expression rate and *d*_*i *_is the degradation rate. Here we use one value *τ*_*ij *_to represent regulatory delays of gene *j *related to the expression of gene *i*. The *cis*-regulatory function *f*_*i*_(*x*_*j*_,..., *x*_*k*_) includes both positive and negative regulations, given by(3)

and  and  are subsets of positive and negative regulations of the total regulation set *R*, respectively.

For each TF, the regulation is realized by(4)

where *m *is the number of DNA-binding site and *n *represents the cooperative binding of the TF. The present model is a more general approach which includes the proposed *cis*-regulatory function model when *n *= 1 [12,13], the Michaelis-Menten function model when *m *= *n *= 1 [11], and the Hill function model when *n *> 1. Based on the structure of TF p53, the transcription of a p53 target gene is represented by(5)

where *x*_*i*_(*t*) is the expression level of gene *i *and *p*(*t*) is the p53 activity at time *t*. Here *δ*_*i *_is an indicator of the feedback regulation, namely *δ*_*i *_= 0 if p53 inhibit the transcription of gene *i *or *δ*_*i *_= 1 if the transcription is induced by p53. The Hill coefficient was chosen to be 4 since p53 is in the form of tetramer as a transcriptional factor [22].

### Prediction of TF activities

It is assumed that a TF regulates the expression of *N *target genes. The proposed mathematical model (2) can be used to infer the activities of the TF from the expression levels of these *N *target genes. To achieve this, we developed a system of *N *differential equations. Each equation of the system follows the model (2) and represents the expression process of a specific gene. This system contains a number of unknown parameters including the kinetic rates (*c*_*i*_, *k*_*i*_, *d*_*i*_, *τ*_*ij*_) (*i *= 1, ..., *N*) together with the TF activities at *M *measurement time points (*t*_1_,..., *t*_*M*_). Using an optimization method such as the genetic algorithm [45], we can search the optimal model parameters to match the expression levels {*x*_*ij*_, *i *= 1,..., *N*, *j *= 1, ..., *M*} of these *N *target genes at *M *measurement time points of the microarray experiments. The estimated TF activities from the optimization method is the prediction of the TF activities.

Specifically, this work used the nonlinear model (5) to predict the p53 activities from a set of five training target genes (*N *= 5). Here a system of five equations, in which each equation follows the same nonlinear model (5) with different parameters, was used to represent the expression of five training genes. The unknown parameters of the system are rate constants (*c*_*i*_, *k*_*i*_, *K*_*i*_, *d*_*i*_, *τ*_*i*_, *δ*_*i*_) (*i *= 1,..., 5) and p53 activities (*p*_*j *_= *p*(*t*_*j*_), *t*_*j *_= 2, 4,..., 12) at six time points. The activities of p53 at other time points will be obtained by the natural spline interpolation. In total, there are 26 unknown parameters in the system and the p53 activities at 6 time points is our final inference result.

We used a MATLAB toolbox of the genetic algorithm [45] to search the optimal values of these 26 parameters. The search space of each parameter is [0, *W*_MAX_] and the values of *W*_MAX _are [5, 5, 5, 2] for [*c*_*i*_, *k*_*i*_, *K*_*i*_, *d*_*i*_]. For p53 activity *p*_*i*_, the values of *W*_MAX _are unit one. After a set of unknown parameters is created by the genetic algorithm, a program developed in MATLAB was used to simulate the nonlinear system of 5 equations and calculate the objective value. The program is described below.

1. Create an individual of p53 activities (*p*_*i*_, *i *= 1,...,6) and regulatory parameters (*c*_*i*_, *k*_*i*_, *K*_*i*_, *d*_*i*_) (*i *= 1, ..., 5) from the genetic algorithm;

2. Use the natural spline interpolation to calculate p53 activities at time points in [0, 12];

3. Solve the system of 5 equations (5) by using the 4-th order classic Runge-Kutta method for each training gene *i *from the initial expression level *u*_*i*0 _(= *x*_*i*0_), and find the simulated levels *u*_*ij*_(*j *= 1,..., 6);

4. Calculate the estimation error of gene *i *as , where *x*_*ij *_is the microarray expression level. Finally, the objective value is .

### Prediction of putative target genes

Using the predicted TF activities in the previous subsection, we can infer the TF-mediated genetic regulation based on the proposed nonlinear model (2). The genetic algorithm can be used here to search the optimal model parameters in functions (3) and (4) to match the expression level of a putative target gene and examine whether the positive or negative regulation in function (3) is more appropriate to present the genetic regulation. All the genes considered will be ranked by the model error that is defined as the difference between the simulated expression levels from model (2) and microarray expression profiles. Genes with smaller model error will be selected as the putative target genes and further research will be carried out for these genes.

Specifically, we used the newly inferred p53 activity in Figure 2B and nonlinear model (5) to infer the genetic regulation of p53 target genes. There are six unknown parameters for each gene's regulation, namely *c*_*i*_, *k*_*i*_, *K*_*i*_, *d*_*i*_, *τ*_*i *_and *δ*_*i*_. The genetic algorithm was used to search for the optimal values of these six parameters. The value of *δ*_*i *_is determined by another parameter *η*_*i *_whose search area is [-1, 1]; and parameter *η*_*i *_indicates either positive (*η*_*i *_> 0, *δ*_*i *_= 1) or negative (*η*_*i *_< 0, *δ*_*i *_= 0) regulation from p53. The time delay *τ*_*i *_is treated as one of the unknown parameter and its value will be searched by the genetic algorithm. Ten estimates (*c*_*ij*_, *k*_*ij*_, *K*_*ij*_, *d*_*ij*_, *τ*_*ij*_, *δ*_*ij*_) (*j *= 1, ..., 10) were obtained from different implementations of the genetic algorithm. From these 10 estimates, we selected the set of parameters that has the smallest estimation error as the final estimate. The following algorithm was developed to estimate the model parameters.

1. Create an individual of the regulatory parameter (*c*_*i*_, *k*_*i*_, *K*_*i*_, *d*_*i*_, *τ*_*i*_, *δ*_*i*_) from the genetic algorithm;

2. Determine the value of *δ*_*i *_in Equation (5). If *η*_*i *_> 0, *δ*_*i *_= 1. Otherwise *δ*_*i *_= 0;

3. Determine the p53 activities based on activities in Figure 2B and the time delay *τ*_*i*_. *p*(*t *- *τ*_*i*_) = 0 (*t *≤ *τ*_*i*_).

4. Simulate model (5) by using the initial level *u*_*i*0_(= *x*_*i*0_) and find the simulated expression levels *u*_*ij *_(*j *= 1,..., *m*);

5. Calculate the objective value .

### Sensitivity analysis

Here we use the Khalil method [27] for sensitivity analysis of mathematical models. For a given model (the base model) with parameter *p*(6)

we consider the solution x* of this system with a perturbed parameter *p *+ Δ*p*. The difference between solutions x* and *x *is

Together with the base model (6), the adjacent model for parameter *p *is(7)

Here *Ep *represents the drift of the solution with a unit parameter perturbation. The solutions of the adjacent models for certain important parameters in the base model give insight into which parameter induces the largest error in solutions and when errors will be the largest in simulations.

## Authors' contributions

TT developed the mathematical model, carried out simulation and inference, and identified p53 target genes. JW analyzed the Microarray expression data, identified p53 target genes and analyzed the binding motif. Both authors wrote the paper and approved the final manuscript.

## Supplementary Material

Additional file 1 **A complete list of 1,312 selected gene probes**. Here we listed the 1,312 most relevant response probes to ionizing radiation based on the selection of Fisher's linear distriminant. These probes were further assigned to 40 clusters according to their expression profiles across all time points. The detailed information of each probe includes Gene title, GO information, nonlinear model estimation errors, gene regulation state, time delay effect, gene regulation state based on event method or correlation methods. Click here for file

Additional file 2 **Detailed information for the top 317 putative p53 target genes**. Here we list the putative p53 target gene information (i.e. AffyProbe ID, gene symbol and gene title), quantitative model estimation error, target gene regulation state inferred by quantitative model (regulate: 1 represents positive regulation by p53 but -1 represents negative regulation by p53), time delay effect (delay: hour), target gene regulation state inferred by event method (event score > 0 represents positive regulation, event sore < 0 represents negative regulation), target gene regulation state inferred by correlation method (correlation coefficient > 0 represents positive regulation, correlation coefficient < 0 represents negative regulation), and the number of motif count of perfect match of 10-mer p53 binding motif on 10 kb upstream region (motif counts). Click here for file

Additional file 3 **Predicted TF affinity profiles for four lists of putative p53 target genes**. Here we used MatrixREDUCE program to compute sequence affinity profiles for 409 human TF weight matrices on four lists of p53 target genes. An average sequence affinity of each TF on each list is listed below according to their prediction methods such as MBDM method, nonlinear quantitative model, microarray gene expression analysis, and Chip-PET analysis, respectively. The corresponding affinity score for random sequences and its associated relative ratio to each list are presented as well. Click here for file

Additional file 4 **Hierarchical clustering of relative sequence affinity ratios**. Here we present results of hierarchical clustering of relative sequence affinity ratios for 409 human TFs across four list of putative p53 target genes. Yellow colour represents enriched TFs but blue colour represents depleted TFs. Click here for file
