# Supplementary material for: Quantitative model for inferring dynamic regulation of the tumour suppressor gene p53
Source: BMC Bioinformatics. 2010 Jan 19;11:36. doi: 10.1186/1471-2105-11-36 (PMC2832896; doi:10.1186/1471-2105-11-36)
Supplement: Additional file 2 — Detailed information for the top 317 putative p53 target genes. Here we list the putative p53 target gene information (i.e. AffyProbe ID, gene symbol and gene title), quantitative model estimation error, target gene regulation state inferred by quantitative model (regulate: 1 represents positive regulation by p53 but -1 represents negative regulation by p53), time delay effect (delay: hour), target gene regulation state inferred by event method (event score > 0 represents positive regulation, event sore < 0 represents negative regulation), target gene regulation state inferred by correlation method (correlation coefficient > 0 represents positive regulation, correlation coefficient < 0 represents negative regulation), and the number of motif count of perfect match of 10-mer p53 binding motif on 10 kb upstream region (motif counts). [file 1471-2105-11-36-S2.PDF]

Quantitative model for inferring dynamic regulation of the tumour suppressor gene p53
Supplementary Information

Junbai Wang and Tianhai Tian

Supplementary Table 2. Detailed information for the top 317 putative p53 target genes.
Here we list the putative p53 target gene information (i.e. AffyProbe ID, gene symbol and gene title), quantitative model estimation error, target gene regulation state inferred by quantitative model (regulation: 1 represents positive regulation by p53 but -1 represents negative regulation by p53), time delay effect (delay: hours), target gene regulation state inferred by event method (event score >0 represents positive regulation, event sore <0 represents negative regulation), target gene regulation state inferred by correlation method (correlation coefficient >0 represents positive regulation, correlation coefficient <0 represents negative regulation), and the number of motif count of perfect match of 10-mer p53 binding motif on 10kb upstream region (motif counts).

	Order	Probe Set ID	Gene Symbol	Gene Title	model estimation error	regulation	delay	event score	correlation coefficient	p-value	motif counts
1	1	217732_S_AT	ITM2B	integral membrane protein 2B	0.0382	1	0	1	0.207	0.656	4
2	2	205347_S_AT	TMSL8	thymosin-like 8	0.0828	1	1.823	3	-0.1556	0.739	6
3	3	211630_S_AT	GSS	glutathione synthetase	0.11	1	0	-6	0.4575	0.302	10
4	4	201202_AT	PCNA	proliferating cell nuclear antigen	0.1148	1	1.07	2	0.6102	0.146	10
5	5	208812_X_AT	HLA-C	major histocompatibility complex, class I, C	0.1216	1	0	2	0.6533	0.112	NA
6	6	202649_X_AT	RPS19	ribosomal protein S19	0.1396	1	1.42	2	0.1243	0.791	12
7	7	211714_X_AT	TUBB	tubulin, beta	0.1495	1	1.403	0	0.5364	0.215	NA
8	9	201761_AT	MTHFD2	methylenetetrahydrofolate dehydrogenase (NADP+ dependent) 2	0.1848	-1	0.39	1	-0.2435	0.599	4
9	10	202605_AT	GUSB	glucuronidase, beta	0.1933	1	0.638	5	0.146	0.755	2
10	11	209140_X_AT	HLA-B	major histocompatibility complex, class I, B	0.1956	1	0.193	3	0.7715	0.042	3
11	12	210968_S_AT	RTN4	reticulon 4	0.1996	-1	0	-3	-0.72	0.068	10
12	13	201476_S_AT	RRM1	ribonucleotide reductase M1 polypeptide	0.2046	1	0.76	0	0.3134	0.494	10
13	14	204026_S_AT	ZWINT	ZW10 interactor	0.2087	1	1.564	3	-0.0447	0.924	8
14	18	216705_S_AT	ADA	adenosine deaminase	0.2235	1	1.683	4	-0.2144	0.644	4
15	20	202503_S_AT	KIAA0101	KIAA0101	0.2318	1	1.191	-4	0.0023	0.996	6
16	21	218740_S_AT	CDK5RAP3	CDK5 regulatory subunit associated protein 3	0.2382	1	0.248	0	0.0714	0.879	4
17	23	213060_S_AT	CHI3L2	chitinase 3-like 2	0.2785	1	0.705	3	0.0297	0.95	12
18	24	221943_X_AT	RPL38	Ribosomal protein L38	0.2858	1	1.44	-2	0.5116	0.241	6
19	25	218883_S_AT	MLF1IP	MLF1 interacting protein	0.2891	1	0.974	0	0.252	0.586	4

20	30	201721_S_AT	LAPTM5	lysosomal associated multispanning membrane protein 5	0.3121	1	1.545	2	-0.0289	0.951	4
21	31	208149_X_AT	DDX11	DEAD/H (Asp-Glu-Ala-Asp/His) box polypeptide 11	0.3408	1	0.146	3	0.6388	0.123	4
22	32	209773_S_AT	RRM2	ribonucleotide reductase M2 polypeptide	0.3454	1	0	0	-0.0744	0.874	4
23	33	218403_AT	TRIAP1	TP53 regulated inhibitor of apoptosis 1	0.35	1	0	2	0.7745	0.041	8
24	36	201577_AT	NME1	non-metastatic cells 1, protein (NM23A)	0.3645	1	1.685	2	0.253	0.584	4
25	38	210774_S_AT	NCOA4	nuclear receptor coactivator 4	0.3703	1	0	0	-0.0418	0.929	12
26	40	221081_S_AT	DENND2D	DENN/MADD domain containing 2D	0.3848	1	2E-04	-1	0.3352	0.462	10
27	41	208998_AT	UCP2	uncoupling protein 2 (mitochondrial, proton carrier)	0.4023	1	0	4	0.4023	0.371	6
28	43	204386_S_AT	MRP63	mitochondrial ribosomal protein 63	0.4127	1	1.876	0	-0.2646	0.566	6
29	47	218140_X_AT	SRPRB	signal recognition particle receptor, B subunit	0.447	-1	1.639	2	0.0962	0.838	8
30	48	218007_S_AT	RPS27L	ribosomal protein S27-like	0.4474	1	4E-04	-1	0.3573	0.431	6
31	50	218870_AT	ARHGAP15	Rho GTPase activating protein 15	0.4526	-1	1.135	2	-0.6821	0.091	4
32	52	205932_S_AT	MSX1	msh homeobox 1	0.4731	1	0	0	-0.064	0.892	2
33	55	221702_S_AT	TM2D3	TM2 domain containing 3	0.485	-1	0.653	6	-0.4115	0.359	8
34	56	201416_AT	SOX4	SRY (sex determining region Y)-box 4	0.4904	1	0	3	-0.1002	0.831	6
35	57	214041_X_AT	RPL37A	Ribosomal protein L37a	0.4914	1	0.296	4	0.7843	0.037	11
36	58	208796_S_AT	CCNG1	cyclin G1	0.5079	1	8E-04	6	0.4262	0.34	4
37	59	201710_AT	MYBL2	v-myb myeloblastosis viral oncogene homolog-like 2	0.5129	1	0	0	0.809	0.028	6
38	60	203695_S_AT	DFNA5	deafness, autosomal dominant 5	0.5244	1	0.497	0	-0.0838	0.858	6
39	62	214022_S_AT	IFITM1	interferon induced transmembrane protein 1 (9-27)	0.5422	-1	0.194	0	-0.6534	0.112	NA
40	63	212414_S_AT	N-PAC /// SEPT6	septin 6 /// cytokine-like nuclear factor n-pac	0.5563	-1	0	1	-0.3801	0.4	NA
41	65	218167_AT	AMZ2	archaemetzincins-2	0.5655	1	0	0	0.4658	0.292	4
42	67	212971_AT	CARS	cysteinyl-tRNA synthetase	0.579	-1	1.378	-3	-0.0587	0.901	8
43	68	201626_AT	INSIG1	insulin induced gene 1	0.5827	-1	0	0	0.1929	0.679	NA
44	69	203554_X_AT	PTTG1	pituitary tumor-transforming 1	0.5878	-1	0	0	-0.8967	0.006	4
45	76	208634_S_AT	MACF1	microtubule-actin crosslinking factor 1	0.6219	1	1.209	-1	0.2987	0.515	4
46	77	201210_AT	DDX3X	DEAD (Asp-Glu-Ala-Asp) box polypeptide 3, X-linked	0.6354	-1	1.755	-2	-0.1888	0.685	4
47	78	203409_AT	DDB2	damage-specific DNA binding protein 2, 48kDa	0.6436	1	0	6	0.5886	0.165	7
48	79	216320_X_AT	MST1	macrophage stimulating 1	0.6562	1	0.916	-1	0.0215	0.964	7.33
49	81	202693_S_AT	STK17A	serine/threonine kinase 17a	0.6612	-1	1.914	0	0.6706	0.099	8
50	82	201478_S_AT	DKC1	dyskeratosis congenita 1, dyskerin	0.6612	-1	1.325	-3	0.0145	0.976	4
51	83	200608_S_AT	RAD21	RAD21 homolog (S. pombe)	0.6737	-1	0	0	-0.9242	0.003	4
52	84	203214_X_AT	CDC2	cell division cycle 2, G1 to S and G2 to M	0.6748	1	1.774	0	-0.5441	0.207	0
53	85	212175_S_AT	AK2	adenylate kinase 2	0.685	-1	0	1	-0.0708	0.88	12
54	87	202431_S_AT	MYC	v-myc myelocytomatosis viral oncogene homolog	0.6894	-1	0	-3	-0.3609	0.426	6
55	88	205961_S_AT	PSIP1	PC4 and SFRS1 interacting protein 1	0.709	-1	0.001	-4	-0.9341	0.002	3
56	89	201222_S_AT	RAD23B	RAD23 homolog B (S. cerevisiae)	0.7108	-1	1.333	4	-0.6191	0.138	2

57	91	203509_AT	SORL1	sortilin-related receptor, L(DLR class) A repeats-containing	0.745	1	1.695	0	0.2524	0.585	8
58	92	215887_AT	ZNF277P	zinc finger protein 277 pseudogene	0.7541	1	0	1	0.0729	0.877	6
59	95	218237_S_AT	SLC38A1	solute carrier family 38, member 1	0.7724	-1	1.043	-2	-0.2515	0.586	6
60	96	212037_AT	PNN	pinin, desmosome associated protein	0.784	-1	1.545	0	-0.1922	0.68	14
61	97	208647_AT	FDFT1	farnesyl-diphosphate farnesyltransferase 1	0.7841	-1	0	2	0.531	0.22	4
62	99	205674_X_AT	FXYD2	FXYD domain containing ion transport regulator 2	0.7918	1	0	0	-0.1412	0.763	5
63	101	212227_X_AT	EIF1	eukaryotic translation initiation factor 1	0.826	-1	0	0	0.3385	0.458	4
64	102	214646_AT	HIST1H3J	Histone cluster 1, H3j	0.8272	1	0.255	0	-0.1378	0.768	6.73
65	106	218499_AT	RP6-213H19.1	serine/threonine protein kinase MST4	0.8586	-1	1.33	2	-0.5911	0.162	8
66	107	201010_S_AT	TXNIP	thioredoxin interacting protein	0.8786	1	2E-04	1	0.0699	0.882	8
67	108	206323_X_AT	OPHN1	oligophrenin 1	0.8791	1	1.841	2	0.3604	0.427	4
68	110	213857_S_AT	CD47	CD47 molecule	0.9055	1	1.462	0	-0.6639	0.104	4
69	111	202580_X_AT	FOXM1	forkhead box M1	0.9156	1	0.725	0	-0.1935	0.678	6
70	112	217294_S_AT	ENO1	enolase 1, (alpha)	0.9282	-1	1.639	0	-0.0816	0.862	0.5
71	113	201410_AT	PLEKHB2	pleckstrin homology domain containing, family B (evectins) member 2	0.9509	-1	1.218	-1	-0.3998	0.374	6
72	118	209166_S_AT	MAN2B1	mannosidase, alpha, class 2B, member 1	0.978	1	4E-04	0	0.703	0.078	8
73	120	201690_S_AT	TPD52	tumor protein D52	0.9861	-1	0	-2	-0.0458	0.922	2
74	123	218732_AT	PTRH2	peptidyl-tRNA hydrolase 2	1.0112	-1	1.494	1	0.1904	0.683	6
75	125	219863_AT	HERC5	hect domain and RLD 5	1.0149	1	0	0	0.3032	0.509	8
76	126	218115_AT	ASF1B	ASF1 anti-silencing function 1 homolog B	1.0202	1	0	-2	0.2747	0.551	4
77	127	201183_S_AT	CHD4	chromodomain helicase DNA binding protein 4	1.028	-1	1.303	0	-0.0729	0.877	4
78	128	207614_S_AT	CUL1	cullin 1	1.037	1	2E-04	-2	0.2006	0.666	10
79	129	201012_AT	ANXA1	annexin A1	1.047	1	6E-04	-1	0.9383	0.002	6
80	132	205692_S_AT	CD38	CD38 molecule	1.0689	1	6E-04	2	0.382	0.398	6
81	133	212185_X_AT	MT2A	metallothionein 2A	1.0698	1	0	0	-0.3508	0.44	8
82	138	208980_S_AT	UBC	ubiquitin C	1.1005	1	0	2	0.8126	0.026	6
83	139	209584_X_AT	APOBEC3C	apolipoprotein B mRNA editing enzyme, catalytic polypeptide-like 3C	1.1158	-1	0	0	0.089	0.849	4
84	141	213204_AT	PARC	p53-associated parkin-like cytoplasmic protein	1.1296	1	0	1	0.1756	0.706	NA
85	143	216620_S_AT	ARHGEF10	Rho guanine nucleotide exchange factor (GEF) 10	1.1367	-1	1.567	-1	0.3526	0.438	4
86	144	218131_S_AT	GATAD2A	GATA zinc finger domain containing 2A	1.141	-1	1.153	-3	-0.0535	0.909	4
87	145	209375_AT	XPC	xeroderma pigmentosum, complementation group C	1.1468	1	0.006	4	0.6337	0.127	0
88	146	212488_AT	COL5A1	collagen, type V, alpha 1	1.1566	1	0.801	0	-0.1657	0.723	4
89	147	201834_AT	PRKAB1	protein kinase, AMP-activated, beta 1 non-catalytic subunit	1.1574	1	0	-2	0.8187	0.024	12
90	150	210074_AT	CTSL2	cathepsin L2	1.1832	1	1.521	0	-0.3079	0.502	8
91	151	207169_X_AT	DDR1	discoidin domain receptor family, member 1	1.1946	-1	1.345	0	0.4795	0.276	NA
92	152	209849_S_AT	RAD51C	RAD51 homolog C (S. cerevisiae)	1.2028	1	6E-04	0	0.3346	0.463	0
93	154	208308_S_AT	GPI	glucose phosphate isomerase	1.2242	-1	0	0	0.3118	0.496	8

94	157	201251_AT	PKM2	pyruvate kinase, muscle	1.2523	-1	0	1	0.2504	0.588	14
95	159	219361_S_AT	ISG20L1	interferon stimulated exonuclease gene 20kDa-like 1	1.2623	1	0	-2	0.8321	0.02	8
96	160	201663_S_AT	SMC4	structural maintenance of chromosomes 4	1.2648	1	0	5	-0.4098	0.361	12
97	161	221434_S_AT	C14orf156	chromosome 14 open reading frame 156	1.2672	-1	0	1	0.3701	0.414	6
98	162	206337_AT	CCR7	chemokine (C-C motif) receptor 7	1.2735	1	9E-04	0	0.2385	0.607	6
99	164	205548_S_AT	BTG3	BTG family, member 3	1.3042	1	0.538	0	0.7656	0.045	12
100	166	396_F_AT	EPOR	erythropoietin receptor	1.3068	1	1.721	3	0.3703	0.414	4
101	170	209685_S_AT	PRKCB1	protein kinase C, beta 1	1.3258	-1	0.718	0	-0.687	0.088	4
102	174	205255_X_AT	TCF7	transcription factor 7 (T-cell specific, HMG-box)	1.3697	1	0	-2	-0.4909	0.263	4
103	178	204958_AT	PLK3	polo-like kinase 3 (Drosophila)	1.4262	-1	1.517	4	0.7745	0.041	4
104	180	207727_S_AT	MUTYH	mutY homolog (E. coli)	1.4345	1	0	0	-0.2684	0.561	12
105	183	209698_AT	CCHCR1	coiled-coil alpha-helical rod protein 1	1.4872	1	1.017	0	0.2605	0.573	20
106	185	205266_AT	LIF	leukemia inhibitory factor	1.5033	1	0.066	0	0.5593	0.192	2
107	186	202718_AT	IGFBP2	insulin-like growth factor binding protein 2, 36kDa	1.5237	-1	0	-3	0.0233	0.96	2
108	187	204825_AT	MELK	maternal embryonic leucine zipper kinase	1.5416	1	0	0	-0.4584	0.301	6
109	188	204759_AT	RCBTB2	regulator of chromosome condensation (RCC1) and BTB (POZ) domain containing protein 2	1.5467	-1	0	0	-0.9148	0.004	4
110	189	216237_S_AT	MCM5	minichromosome maintenance complex component 5	1.5526	1	0	1	0.7149	0.071	0
111	190	208152_S_AT	DDX21	DEAD (Asp-Glu-Ala-Asp) box polypeptide 21	1.5638	-1	0	0	0.383	0.397	6
112	191	218690_AT	PDLIM4	PDZ and LIM domain 4	1.5669	1	0.707	-3	0.0219	0.963	4
113	192	209974_S_AT	BUB3	BUB3 budding uninhibited by benzimidazoles 3	1.5724	-1	2E-04	3	-0.8857	0.008	4
114	193	207121_S_AT	MAPK6	mitogen-activated protein kinase 6	1.6002	-1	1.617	-2	0.1758	0.706	3.33
115	196	212021_S_AT	MKI67	antigen identified by monoclonal antibody Ki-67	1.6036	1	1.704	-2	-0.6542	0.111	2
116	197	211615_S_AT	LRPPRC	leucine-rich PPR-motif containing	1.6042	-1	0	-1	0.0943	0.841	6
117	202	202729_S_AT	LTBP1	latent transforming growth factor beta binding protein 1	1.6431	1	0.609	1	-0.0343	0.942	4
118	203	213293_S_AT	TRIM22	tripartite motif-containing 22	1.6431	1	0	0	0.5683	0.183	6
119	205	204321_AT	NEO1	neogenin homolog 1 (chicken)	1.6711	1	0	2	0.3467	0.446	6
120	207	220547_S_AT	FAM35A	family with sequence similarity 35, member A	1.7078	-1	0	4	0.0181	0.969	6.67
121	210	213039_AT	ARHGEF18	rho/rac guanine nucleotide exchange factor (GEF) 18	1.7194	-1	0	-4	-0.9647	4E-04	6
122	211	212048_S_AT	YARS	tyrosyl-tRNA synthetase	1.7223	-1	0	-3	0.2746	0.551	6
123	212	206792_X_AT	PDE4C	phosphodiesterase 4C, cAMP-specific	1.7234	1	0	3	-0.4213	0.347	4
124	213	204905_S_AT	EEF1E1	eukaryotic translation elongation factor 1 epsilon 1	1.7318	-1	0	0	-0.143	0.76	8
125	214	206445_S_AT	PRMT1	protein arginine methyltransferase 1	1.7375	-1	0	0	0.4594	0.3	2
126	215	205043_AT	CFTR	cystic fibrosis transmembrane conductance regulator (ATP-binding cassette sub-family C, member 7)	1.7426	1	1.66	-4	-0.01	0.983	8
127	216	62987_R_AT	CACNG4	calcium channel, voltage-dependent, gamma subunit 4	1.7512	1	1.958	0	0.2812	0.541	4
128	218	208690_S_AT	PDLIM1	PDZ and LIM domain 1 (elfin)	1.7723	1	1.726	0	0.0842	0.858	10
129	219	217716_S_AT	SEC61A1	Sec61 alpha 1 subunit (S. cerevisiae)	1.7857	1	0	4	0.9787	1E-04	10
130	220	200751_S_AT	HNRNPC	heterogeneous nuclear ribonucleoprotein C (C1/C2)	1.7902	-1	0	-1	-0.0208	0.965	2.67

131	221	218805_AT	GIMAP5	GTPase, IMAP family member 5	1.7907	1	1.906	-6	-0.4229	0.345	8
132	222	206907_AT	TNFSF9	tumor necrosis factor (ligand) superfamily, member 9	1.8037	-1	1.733	6	0.7964	0.032	4
133	223	200075_S_AT	GUK1	guanylate kinase 1	1.8114	-1	1.652	-4	-0.1704	0.715	8
134	226	217833_AT	SYNCRIP	synaptotagmin binding, cytoplasmic RNA interacting protein	1.8335	-1	0	-2	0.0827	0.86	7
135	233	212290_AT	SLC7A1	solute carrier family 7, member 1	1.9201	-1	0	0	0.0172	0.971	8
136	234	213523_AT	CCNE1	cyclin E1	1.9631	1	1.909	-3	0.56	0.191	4
137	235	204951_AT	RHOH	ras homolog gene family, member H	1.9783	1	0	0	-0.8423	0.017	2
138	236	210313_AT	LILRA4	leukocyte immunoglobulin-like receptor, subfamily A (with TM domain), member 4	1.9971	1	0	0	0.3814	0.399	6
139	237	205436_S_AT	H2AFX	H2A histone family, member X	2.0111	1	0	0	-0.6173	0.14	NA
140	239	203747_AT	AQP3	aquaporin 3 (Gill blood group)	2.0187	1	0	-1	0.3084	0.501	4
141	240	221718_S_AT	AKAP13	A kinase (PRKA) anchor protein 13	2.0252	1	0	1	-0.5832	0.169	8
142	241	200731_S_AT	PTP4A1	protein tyrosine phosphatase type IVA, member 1	2.0412	-1	1.771	6	0.7896	0.035	8
143	244	218611_AT	IER5	immediate early response 5	2.0564	-1	1.535	-4	0.5312	0.22	4
144	245	211967_AT	TMEM123	transmembrane protein 123	2.0766	-1	1.683	6	-0.3681	0.417	8
145	248	209608_S_AT	ACAT2	acetyl-Coenzyme A acetyltransferase 2	2.1007	-1	0	-3	0.6509	0.113	4
146	249	220147_S_AT	FAM60A	family with sequence similarity 60, member A	2.101	1	0	1	0.5567	0.194	6
147	251	200648_S_AT	GLUL	glutamate-ammonia ligase (glutamine synthetase)	2.1355	-1	0	-2	-0.307	0.503	6
148	254	200662_S_AT	TOMM20	translocase of outer mitochondrial membrane 20	2.1676	-1	0	6	-0.0638	0.892	10
149	257	202284_S_AT	CDKN1A	cyclin-dependent kinase inhibitor 1A (p21, Cip1)	2.192	1	9E-04	2	0.8018	0.03	12
150	259	201332_S_AT	STAT6	signal transducer and activator of transcription 6, interleukin-4 induced	2.2083	-1	0.918	-1	0.8001	0.031	4
151	260	210582_S_AT	LIMK2	LIM domain kinase 2	2.2208	1	0.441	3	0.4873	0.267	7
152	261	205967_AT	HIST1H4C	histone cluster 1, H4c	2.2236	1	1.783	0	-0.3866	0.392	5.67
153	262	211235_S_AT	ESR1	estrogen receptor 1	2.2253	1	0	0	0.3682	0.416	6
154	263	208983_S_AT	PECAM1	platelet/endothelial cell adhesion molecule	2.2255	1	1.968	-1	-0.5058	0.247	NA
155	265	210387_AT	HIST1H2BG	histone cluster 1, H2bg	2.239	1	0	2	-0.2001	0.667	3.8
156	266	202303_X_AT	SMARCA5	SWI/SNF related, matrix associated, actin dependent regulator of chromatin, subfamily a, member 5	2.2479	-1	1.625	1	-0.5731	0.179	8
157	267	211814_S_AT	CCNE2	cyclin E2	2.2559	1	0.843	0	0.6715	0.099	2
158	268	208332_AT	PRY	PTPN13-like, Y-linked	2.2563	1	0	2	0.3293	0.471	10
159	271	218039_AT	NUSAP1	nucleolar and spindle associated protein 1	2.279	1	0	0	-0.6561	0.11	4
160	273	220714_AT	PRDM14	PR domain containing 14	2.2866	1	0.189	-1	0.2132	0.646	8
161	274	209062_X_AT	NCOA3	nuclear receptor coactivator 3	2.2874	1	0	1	0.2883	0.531	6
162	275	208478_S_AT	BAX	BCL2-associated X protein	2.3018	1	0	2	0.0334	0.943	8
163	276	204574_S_AT	MMP19	matrix metallopeptidase 19	2.3081	1	7E-04	-2	0.1933	0.678	6
164	277	202095_S_AT	BIRC5	baculoviral IAP repeat-containing 5 (survivin)	2.3172	-1	0	0	-0.8647	0.012	0
165	278	208591_S_AT	PDE3B	phosphodiesterase 3B, cGMP-inhibited	2.3207	1	0.534	0	-0.2256	0.627	2
166	279	204009_S_AT	KRAS	v-Ki-ras2 Kirsten rat sarcoma viral oncogene homolog	2.3263	-1	0	0	-0.878	0.009	4

167	283	204170_S_AT	CKS2	CDC28 protein kinase regulatory subunit 2	2.3519	-1	0.216	-3	-0.9305	0.002	6
168	284	218251_AT	MID1IP1	MID1 interacting protein 1	2.3678	-1	1.303	-2	0.1475	0.752	6
169	285	209786_AT	HMGN4	high mobility group nucleosomal binding domain 4	2.3802	-1	1.055	4	-0.7378	0.058	6
170	286	201475_X_AT	MARS	methionyl-tRNA synthetase	2.389	1	0	0	0.7356	0.06	2
171	287	210213_S_AT	EIF6	eukaryotic translation initiation factor 6	2.3989	-1	0	2	0.3691	0.415	12
172	289	205569_AT	LAMP3	lysosomal-associated membrane protein 3	2.4068	-1	0	0	0.5024	0.251	6
173	290	203752_S_AT	JUND	jun D proto-oncogene	2.4195	1	0	0	0.6247	0.134	0
174	292	211005_AT	LAT	linker for activation of T cells	2.4326	1	0	0	-0.8319	0.02	4
175	293	217051_S_AT	SS18	synovial sarcoma translocation, chromosome 18	2.4477	1	0.247	2	0.1755	0.707	6
176	294	206798_X_AT	DLEC1	deleted in lung and esophageal cancer 1	2.482	1	0.724	-3	0.3512	0.44	6
177	296	221404_AT	IL1F6	interleukin 1 family, member 6 (epsilon)	2.4864	1	3E-04	-3	0.4739	0.283	2
178	301	203806_S_AT	FANCA	Fanconi anemia, complementation group A	2.5067	1	0	-3	0.6271	0.132	12
179	302	212252_AT	CAMKK2	calcium/calmodulin-dependent protein kinase kinase 2, beta	2.5227	-1	1.687	-4	0.2518	0.586	4
180	305	207348_S_AT	LIG3	ligase III, DNA, ATP-dependent	2.5334	-1	1.729	-2	0.8643	0.012	6
181	306	217373_X_AT	MDM2	Mdm2, transformed 3T3 cell double minute 2, p53 binding protein (mouse)	2.5517	-1	0	1	0.7957	0.032	2
182	309	211725_S_AT	BID	BH3 interacting domain death agonist	2.5805	-1	0	1	0.0957	0.838	6
183	311	206662_AT	GLRX	glutaredoxin (thioltransferase)	2.5968	-1	0	0	-0.917	0.004	8
184	312	203725_AT	GADD45A	growth arrest and DNA-damage-inducible, alpha	2.6107	1	0	0	0.761	0.047	2
185	313	204529_S_AT	TOX	thymocyte selection-associated high mobility group box	2.6176	-1	0	0	-0.7998	0.031	6
186	314	213288_AT	MBOAT2	membrane bound O-acyltransferase domain containing 2	2.6226	1	1.089	2	-0.1771	0.704	6
187	317	219752_AT	RASAL1	RAS protein activator like 1 (GAP1 like)	2.6483	1	0.431	-2	0.2934	0.523	2
188	318	203580_S_AT	SLC7A6	solute carrier family 7, member 6	2.658	1	0	0	0.6904	0.086	8
189	319	202827_S_AT	MMP14	matrix metallopeptidase 14 (membrane-inserted)	2.6722	1	0	0	0.4555	0.304	4
190	321	204213_AT	PIGR	polymeric immunoglobulin receptor	2.6813	1	0	-1	0.2851	0.535	10
191	322	209736_AT	SOX13	SRY (sex determining region Y)-box 13	2.6878	1	0	0	0.4174	0.352	8
192	326	221701_S_AT	STRA6	stimulated by retinoic acid gene 6 homolog (mouse)	2.6974	1	0	3	-0.6688	0.101	8
193	328	31861_AT	IGHMBP2	immunoglobulin mu binding protein 2	2.7142	1	0	-2	0.267	0.563	8
194	329	206892_AT	AMHR2	anti-Mullerian hormone receptor, type II	2.738	1	0	2	0.3873	0.391	10
195	333	201804_X_AT	TBCB	tubulin folding cofactor B	2.7736	-1	0	-1	0.0442	0.925	12
196	334	217033_X_AT	NTRK3	neurotrophic tyrosine kinase, receptor, type 3	2.7737	1	6E-04	1	0.3274	0.474	6
197	335	205813_S_AT	MAT1A	methionine adenosyltransferase I, alpha	2.7742	1	0	1	0.3883	0.389	6
198	339	211682_X_AT	UGT2B28	UDP glucuronosyltransferase 2 family, polypeptide B28	2.7984	1	0	3	0.2354	0.611	10
199	340	210586_X_AT	RHD	Rh blood group, D antigen	2.8076	1	0	2	0.1355	0.772	7.33
200	344	202466_AT	POLS	polymerase (DNA directed) sigma	2.8305	1	0	2	-0.6615	0.106	8
201	345	206217_AT	EDA	ectodysplasin A	2.8346	1	0.446	-3	0.2185	0.638	6
202	348	200837_AT	BCAP31	B-cell receptor-associated protein 31	2.8463	-1	1.619	-2	-0.262	0.57	10
203	350	207524_AT	ST7	suppression of tumorigenicity 7	2.8716	1	0	-1	0.2873	0.532	2

204	352	209083_AT	CORO1A	coronin, actin binding protein, 1A	2.8759	-1	0	2	0.1073	0.819	2
205	353	215416_S_AT	STOML2	stomatin (EPB72)-like 2	2.8871	-1	0	2	0.5201	0.232	6
206	358	211040_X_AT	GTSE1	G-2 and S-phase expressed 1	2.9365	1	0	0	-0.6074	0.148	4
207	360	206681_X_AT	GP2	glycoprotein 2 (zymogen granule membrane)	2.9659	1	0	-1	0.2718	0.555	10
208	364	201853_S_AT	CDC25B	cell division cycle 25 homolog B (S. pombe)	2.9981	-1	0	0	-0.7383	0.058	6
209	366	210577_AT	CASR	calcium-sensing receptor	3.0246	1	0	-3	0.3618	0.425	6
210	368	204178_S_AT	RBM14	RNA binding motif protein 14	3.0801	1	0	2	0.8619	0.013	4
211	369	202963_AT	RFX5	regulatory factor X, 5	3.0867	1	0	0	0.5331	0.218	6
212	370	214448_X_AT	NFKBIB	nuclear factor of kappa light polypeptide gene enhancer in B-cells inhibitor, beta	3.0975	-1	0	-2	0.6892	0.087	8
213	372	215543_S_AT	LARGE	like-glycosyltransferase	3.1037	1	0.248	-3	0.2355	0.611	6
214	373	209814_AT	ZNF330	zinc finger protein 330	3.1067	-1	0	-1	-0.1931	0.678	6
215	380	210405_X_AT	TNFRSF10B	tumor necrosis factor receptor superfamily, member 10b	3.1484	-1	0	-3	0.7397	0.057	0
216	381	204401_AT	KCNN4	potassium intermediate/small conductance calcium-activated channel, subfamily N, member 4	3.1529	1	2E-04	0	0.3392	0.457	6
217	383	204197_S_AT	RUNX3	runt-related transcription factor 3	3.1638	-1	0.688	0	0.8256	0.022	12
218	385	201516_AT	SRM	spermidine synthase	3.171	1	0	0	0.5826	0.17	10
219	386	207644_AT	FOXH1	forkhead box H1	3.1754	1	0.662	-2	0.1014	0.829	6
220	387	38149_AT	ARHGAP25	Rho GTPase activating protein 25	3.1883	1	0.556	0	-0.2422	0.601	6
221	388	216356_X_AT	BAIAP3	BAI1-associated protein 3	3.1903	1	0	-3	0.5166	0.235	6
222	390	209626_S_AT	OSBPL3	oxysterol binding protein-like 3	3.2098	1	5E-04	4	0.3433	0.451	18
223	391	221407_AT	GJA9	gap junction protein, alpha 9, 36kDa	3.2126	1	7E-04	0	0.4763	0.28	4
224	392	203476_AT	TPBG	trophoblast glycoprotein	3.2153	1	0	6	-0.5657	0.186	4
225	394	201292_AT	TOP2A	topoisomerase (DNA) II alpha 170kDa	3.2295	-1	0	0	-0.7684	0.044	6
226	398	207669_AT	KRT83	keratin 83	3.2388	1	0	-3	0.4425	0.32	4
227	399	202504_AT	TRIM29	tripartite motif-containing 29	3.2617	1	0	1	0.4337	0.331	10
228	401	206508_AT	CD70	CD70 molecule	3.2774	-1	0	6	0.681	0.092	8
229	404	202652_AT	APBB1	amyloid beta (A4) precursor protein-binding, family B, member 1 (Fe65)	3.2901	1	0.527	-3	0.1727	0.711	4
230	405	213520_AT	RECQL4	RecQ protein-like 4	3.2939	1	0	-3	0.1501	0.748	4
231	410	201508_AT	IGFBP4	insulin-like growth factor binding protein 4	3.3229	1	0.172	2	0.1975	0.671	2
232	413	212218_S_AT	FASN	fatty acid synthase	3.3316	1	0	-1	0.4701	0.287	6
233	414	65517_AT	AP1M2	adaptor-related protein complex 1, mu 2 subunit	3.3355	1	0	-1	0.3989	0.375	6
234	417	208189_S_AT	MYO7A	myosin VIIA	3.3521	1	0	-1	0.2458	0.595	10
235	418	218708_AT	NXT1	NTF2-like export factor 1	3.3559	1	0	4	0.3664	0.419	4
236	422	220379_AT	FSCN3	fascin homolog 3, actin-bundling protein, testicular	3.3855	1	0	-2	0.2638	0.568	8
237	424	202887_S_AT	DDIT4	DNA-damage-inducible transcript 4	3.3989	1	0	-2	-0.2085	0.654	8
238	426	214805_AT	EIF4A1	Eukaryotic translation initiation factor 4A, isoform 1	3.4231	1	0	0	-0.3179	0.487	8.5
239	427	204620_S_AT	VCAN	versican	3.4235	1	1E-04	2	0.3856	0.393	6

240	430	209714_S_AT	CDKN3	cyclin-dependent kinase inhibitor 3	3.4984	-1	0	-2	-0.9869	0	4
241	431	209324_S_AT	RGS16	regulator of G-protein signaling 16	3.5064	-1	0.864	-2	0.7215	0.067	6
242	436	202585_S_AT	NFX1	nuclear transcription factor, X-box binding 1	3.5509	-1	0	1	0.7689	0.043	8
243	438	221478_AT	BNIP3L	BCL2/adenovirus E1B 19kDa interacting protein 3-like	3.5548	-1	1.777	0	-0.4907	0.264	8
244	440	205291_AT	IL2RB	interleukin 2 receptor, beta	3.5702	1	0	-2	0.2766	0.548	8
245	443	213727_X_AT	MPPE1	metallophosphoesterase 1	3.5961	1	1.486	0	-0.0979	0.835	4
246	444	203712_AT	KIAA0020	KIAA0020	3.5976	-1	0	2	0.4834	0.272	6
247	448	218219_S_AT	LANCL2	LanC lantibiotic synthetase component C-like 2	3.6428	-1	1.301	-2	0.885	0.008	10
248	450	210052_S_AT	TPX2	TPX2, microtubule-associated, homolog	3.6494	-1	0	0	-0.731	0.062	4
249	451	221640_S_AT	LRDD	leucine-rich repeats and death domain containing	3.6573	1	0	4	0.4923	0.262	NA
250	452	201953_AT	CIB1	calcium and integrin binding 1 (calmyrin)	3.6638	-1	0	0	-0.1189	0.8	2
251	454	218508_AT	DCP1A	DCP1 decapping enzyme homolog A	3.6753	1	0	2	0.7213	0.067	12
252	455	203045_AT	NINJ1	ninjurin 1	3.6756	-1	0.521	-4	0.7117	0.073	8
253	457	216153_X_AT	RECK	reversion-inducing-cysteine-rich protein with kazal motifs	3.6788	1	0	6	0.3037	0.508	4
254	458	203760_S_AT	SLA	Src-like-adaptor	3.6823	1	0.594	1	-0.15	0.748	6
255	460	211961_S_AT	RAB7A	RAB7A, member RAS oncogene family	3.6833	-1	0.582	-2	-0.3706	0.413	4
256	461	217985_S_AT	BAZ1A	bromodomain adjacent to zinc finger domain, 1A	3.6862	-1	0	0	-0.3025	0.51	4
257	462	209239_AT	NFKB1	nuclear factor of kappa light polypeptide gene enhancer in B-cells 1 (p105)	3.6882	-1	1.122	0	0.7783	0.039	6
258	469	211497_X_AT	NKX3-1	NK3 homeobox 1	3.7467	1	0	2	0.4343	0.33	2
259	472	203755_AT	BUB1B	BUB1 budding uninhibited by benzimidazoles 1 homolog beta (yeast)	3.7625	1	0	0	-0.6966	0.082	4
260	476	207537_AT	PFKFB1	6-phosphofructo-2-kinase/fructose-2,6-biphosphatase 1	3.8235	1	5E-04	2	0.4992	0.254	12
261	478	203418_AT	CCNA2	cyclin A2	3.8462	1	0	0	-0.7477	0.053	4
262	479	217001_X_AT	HLA-DOA	major histocompatibility complex, class II, DO alpha	3.8545	1	8E-04	2	0.4543	0.306	14.7
263	482	218009_S_AT	PRC1	protein regulator of cytokinesis 1	3.8731	1	0	0	-0.7422	0.056	2
264	489	213931_AT	ID2 /// ID2B	inhibitor of DNA binding 2, dominant negative helix-loop-helix protein /// inhibitor of DNA binding 2B, dominant negative helix-loop-helix protein	3.9418	1	0	5	0.1243	0.791	NA
265	492	201465_S_AT	JUN	jun oncogene	3.9493	-1	0.496	0	0.214	0.645	10
266	500	218346_S_AT	SESN1	sestrin 1	4.0085	1	0.001	-4	0.7022	0.079	2
267	501	212457_AT	TFE3	transcription factor binding to IGHM enhancer 3	4.0232	-1	0.835	4	0.6712	0.099	4
268	504	207628_S_AT	WBSCR22	Williams Beuren syndrome chromosome region 22	4.0363	-1	0	0	0.1793	0.701	12
269	505	210221_AT	CHRNA3	cholinergic receptor, nicotinic, alpha 3	4.0572	1	1.681	-6	-0.4362	0.328	8
270	507	211475_S_AT	BAG1	BCL2-associated athanogene	4.0795	1	0	-2	0.7451	0.055	4
271	508	221486_AT	ENSA	endosulfine alpha	4.0835	-1	0.345	-2	0.5567	0.194	4
272	514	208519_X_AT	GNRH2	gonadotropin-releasing hormone 2	4.1084	1	0.756	-1	0.4441	0.318	8
273	515	214011_S_AT	HSPC111	hypothetical protein HSPC111	4.1151	-1	0	0	0.0245	0.958	8
274	516	201923_AT	PRDX4	peroxiredoxin 4	4.1214	-1	0	-2	0.1185	0.8	4

275	525	209568_S_AT	RGL1	ral guanine nucleotide dissociation stimulator-like 1	4.1663	1	0	4	0.5761	0.176	8
276	528	208985_S_AT	EIF3J	eukaryotic translation initiation factor 3, subunit J	4.1754	-1	1.553	1	-0.1093	0.816	6
277	532	217850_AT	GNL3	guanine nucleotide binding protein-like 3 (nucleolar)	4.2014	-1	0	0	0.2553	0.581	6
278	533	203007_X_AT	LYPLA1	lysophospholipase I	4.2022	-1	0	-1	-0.2771	0.547	6
279	537	209917_S_AT	TP53AP1	TP53 activated protein 1	4.2446	1	0	0	0.116	0.804	4
280	538	210038_AT	PRKCQ	protein kinase C, theta	4.2448	1	0	-6	-0.8701	0.011	2
281	544	201924_AT	AFF1	AF4/FMR2 family, member 1	4.287	-1	0	-4	-0.9628	5E-04	12
282	545	200749_AT	RAN	RAN, member RAS oncogene family	4.2938	-1	1.255	2	-0.1911	0.682	2
283	546	201802_AT	SLC29A1	solute carrier family 29, member 1	4.3085	1	0	0	0.8428	0.017	10
284	551	207035_AT	SLC30A3	solute carrier family 30 (zinc transporter), member 3	4.3869	1	0	0	0.0426	0.928	6
285	552	205902_AT	KCNN3	potassium intermediate/small conductance calcium-activated channel, subfamily N, member 3	4.3876	1	5E-04	2	0.3649	0.421	8
286	560	207813_S_AT	FDXR	ferredoxin reductase	4.4911	1	1.465	-4	0.6129	0.143	4
287	561	221073_S_AT	NOD1	nucleotide-binding oligomerization domain containing 1	4.4941	1	1.478	-3	0.3983	0.376	8
288	562	202934_AT	HK2	hexokinase 2	4.496	-1	0	-2	-0.1997	0.668	4
289	563	208066_S_AT	GTF2B	general transcription factor IIB	4.5054	1	0	-1	0.7836	0.037	4
290	569	208490_X_AT	HIST1H2BF	histone cluster 1, H2bf	4.5726	1	0	4	-0.4786	0.277	3.8
291	571	218168_S_AT	CABC1	chaperone, ABC1 activity of bc1 complex homolog	4.5767	1	0	0	0.8632	0.012	2
292	572	209646_X_AT	ALDH1B1	aldehyde dehydrogenase 1 family, member B1	4.5778	1	0	4	0.4176	0.351	6
293	574	212530_AT	NEK7	NIMA-related kinase 7	4.6148	-1	0	0	-0.7275	0.064	8
294	576	212638_S_AT	WWP1	WW domain containing E3 ubiquitin protein ligase 1	4.6344	1	1.24	-2	0.1319	0.778	12
295	578	202704_AT	TOB1	transducer of ERBB2, 1	4.6541	1	0	4	0.4192	0.349	4
296	582	212871_AT	MAPKAPK5	mitogen-activated protein kinase-activated protein kinase 5	4.6664	-1	0	-1	0.4797	0.276	8
297	585	204947_AT	E2F1	E2F transcription factor 1	4.7037	1	0	0	0.8586	0.013	8
298	586	211410_X_AT	KIR2DL5A	killer cell immunoglobulin-like receptor, two domains, long cytoplasmic tail, 5A	4.7292	1	0	1	0.3033	0.508	8
299	587	202705_AT	CCNB2	cyclin B2	4.7316	-1	0	0	-0.9412	0.002	8
300	589	216396_S_AT	EI24	etoposide induced 2.4 mRNA	4.765	1	0	6	0.8026	0.03	12
301	593	203140_AT	BCL6	B-cell CLL/lymphoma 6	4.8551	1	1.93	-4	0.0588	0.9	4
302	596	222062_AT	IL27RA	interleukin 27 receptor, alpha	4.8653	-1	0	2	0.488	0.267	4
303	597	201146_AT	NFE2L2	nuclear factor (erythroid-derived 2)-like 2	4.8836	-1	0.385	0	-0.5983	0.156	4
304	598	219103_AT	DDEFL1	development and differentiation enhancing factor-like 1	4.9036	1	0.185	1	0.0992	0.832	8
305	602	204794_AT	DUSP2	dual specificity phosphatase 2	4.9511	-1	1.162	-4	-0.2932	0.523	6
306	603	210439_AT	ICOS	inducible T-cell co-stimulator	4.9664	-1	1.158	2	0.6042	0.151	10
307	610	201746_AT	TP53	tumor protein p53 (Li-Fraumeni syndrome)	5.0265	-1	0.504	-4	0.5769	0.175	0
308	615	218751_S_AT	FBXW7	F-box and WD repeat domain containing 7	5.0622	1	0	-1	0.8541	0.014	4
309	624	201329_S_AT	ETS2	v-ets erythroblastosis virus E26 oncogene homolog 2	5.0995	-1	0.178	-4	0.3903	0.387	10
310	626	201379_S_AT	TPD52L2	tumor protein D52-like 2	5.134	-1	0	-2	0.1342	0.774	10
311	634	218239_S_AT	GTPBP4	GTP binding protein 4	5.2079	-1	0	0	-0.2943	0.522	10

312	638	205780_AT	BIK	BCL2-interacting killer (apoptosis-inducing)	5.2295	1	0	-4	0.7607	0.047	6
313	643	207826_S_AT	ID3	inhibitor of DNA binding 3	5.2733	1	0	4	-0.6606	0.106	8
314	644	208070_S_AT	REV3L	REV3-like, catalytic subunit of DNA polymerase zeta	5.2862	1	0	0	0.5224	0.229	2
315	645	208877_AT	PAK2	p21 (CDKN1A)-activated kinase 2	5.2996	-1	1.73	0	-0.316	0.49	6
316	653	211135_X_AT	LILRB3	leukocyte immunoglobulin-like receptor, subfamily B, member 3	5.4181	1	0	2	0.3641	0.422	3
317	654	218501_AT	ARHGEF3	Rho guanine nucleotide exchange factor 3	5.4288	1	3E-04	-3	0.2315	0.617	12
