# Supplementary material for: Quantitative model for inferring dynamic regulation of the tumour suppressor gene p53
Source: BMC Bioinformatics. 2010 Jan 19;11:36. doi: 10.1186/1471-2105-11-36 (PMC2832896; doi:10.1186/1471-2105-11-36)
Supplement: Additional file 3 — Predicted TF affinity profiles for four lists of putative p53 target genes. Here we used MatrixREDUCE program to compute sequence affinity profiles for 409 human TF weight matrices on four lists of p53 target genes. An average sequence affinity of each TF on each list is listed below according to their prediction methods such as MBDM method, nonlinear quantitative model, microarray gene expression analysis, and Chip-PET analysis, respectively. The corresponding affinity score for random sequences and its associated relative ratio to each list are presented as well. [file 1471-2105-11-36-S3.PDF]

Quantitative Model for Inferring Dynamic Regulation of the Tumour Suppressor Gene p53
Supplementary Information

Junbai Wang and Tianhai Tian

Supplementary Table 3. Predicted TF affinity profiles for four lists of putative p53 target genes.
Here we used MatrixREDUCE program to compute sequence affinity profiles for ~409 human TF weight matrices on four lists of p53 target genes. An average sequence affinity of each TF on each list is listed below according to their prediction methods such as MBDM method, nonlinear quantitative model, microarray gene expression analysis, and Chip-PET analysis, respectively. The corresponding affinity score for random sequences and its associated relative ratio to each list are presented as well.

	MVDM method	Nonlinear quantitative model	Microarray gene expression analysis	Chip-PET analysis	Random sequences	MVDM method (Relative Ratio)	Nonlinear quantitative model (Relative Ratio)	Microarray gene expression analysis (Relative Ratio)	Chip-PET analysis (Relative Ratio)
M00002_:E47	-1.5798	-1.4782	-1.3848	-1.3983	-1.8195	0	0	0	0
M00005_:AP-4	-2.1883	-2.1749	-2.1426	-2.1885	-2.4034	0	0	0	0
M00006_:MEF-2	-4.0538	-4.0216	-4.0133	-4.0311	-4.7168	0	0	0	0
M00007_:ELK-1	0.060953	0.041836	0.056746	0.047053	0.051161	0.009792	-0.0093248	0.0055854	-0.0041074
M00008_:SP1	1.4879	1.5168	1.5528	1.476	1.1182	0.36554	0.39339	0.42792	0.35407
M00017_:ATF	-2.5317	-2.5588	-2.4419	-2.6154	-1.6852	0	0	0	0
M00024_:E2F	-2.4781	-2.4812	-2.5428	-2.5357	-2.067	0	0	0	0
M00025_:ELK-1	-1.5362	-1.4642	-1.4199	-1.4864	-1.0648	0	0	0	0
M00026_:RSRFC4	-5.2969	-5.1548	-5.1006	-4.8756	-6.2048	0	0	0	0
M00033_:P300	0.75253	0.79068	0.78355	0.80219	0.59175	0.16043	0.19827	0.19121	0.20966
M00034_:P53	-11.3134	-12.9991	-14.1177	-8.7678	-12.9599	0	0	0	0
M00037_:NF-E2	-0.92119	-0.83698	-0.83608	-0.82119	-0.95097	0	0	0	0
M00039_:CREB	-0.50172	-0.55211	-0.59629	-0.53937	0.10029	-0.10021	-0.10021	-0.10021	-0.10021
M00040_:CRE-BP1	-2.0471	-1.8342	-2.0668	-1.7861	-0.91127	0	0	0	0
M00041_:CRE-BP1:C-JUN	-1.061	-0.92922	-1.0017	-1.0056	-0.3979	0	0	0	0
M00045_:E4BP4	-2.2667	-2.3795	-2.3334	-2.3064	-2.2005	0	0	0	0
M00050_:E2F	-0.22835	-0.072336	-0.18903	-0.14659	0.6453	-0.6238	-0.6238	-0.6238	-0.6238
M00051_:NF-KAPPAB:(P50)	-1.7974	-2.0038	-2.0173	-1.9539	-2.0721	0	0	0	0
M00052_:NF-KAPPAB:(P65)	-1.4384	-1.2553	-1.1067	-1.2867	-1.36	0	0	0	0
M00053_:C-REL	0.078117	0.15451	0.24285	0.20212	0.13126	-0.05313	0.023246	0.11147	0.070833

M00054_:NF-KAPPAB	-1.5434	-1.457	-1.3277	-1.4978	-1.6306	0	0	0	0
M00056_:MYOGENIN:/:NF-1	-10.345	-10.4021	-10.489	-10.5706	-10.3989	0	0	0	0
M00059_:YY1	0.033688	0.016687	-0.041994	0.03501	-0.22368	0.033685	0.016686	0	0.035007
M00062_:IRF-1	-3.3873	-3.5366	-3.5287	-3.713	-4.4633	0	0	0	0
M00065_:TAL-1BETA:E47	-4.5767	-4.6817	-4.651	-4.4416	-4.6803	0	0	0	0
M00066_:TAL-1ALPHA:E47	-4.7444	-4.5669	-4.7335	-4.7942	-4.7896	0	0	0	0
M00069_:YY1	-5.0017	-4.8382	-4.865	-4.8435	-5.1362	0	0	0	0
M00070_:TAL-1BETA:ITF-2	-4.7488	-5.1524	-5.0834	-4.4669	-5.2767	0	0	0	0
M00071_:E47	-3.4779	-3.5482	-3.2988	-3.5729	-4.2348	0	0	0	0
M00076_:GATA-2	2.1588	2.1776	2.1725	2.187	2.5415	-0.37809	-0.35991	-0.36487	-0.35079
M00077_:GATA-3	1.6504	1.6705	1.6583	1.682	1.7028	-0.052397	-0.03229	-0.044461	-0.020794
M00083_:MZF1	1.9501	1.91	2.0239	1.8937	1.5835	0.36254	0.32365	0.43346	0.30771
M00084_:MZF1	-0.47284	-0.40267	-0.2961	-0.40411	-0.8556	0	0	0	0
M00085_:ZID	-3.4522	-3.4068	-3.5399	-3.4662	-3.5766	0	0	0	0
M00095_:CDP	-4.5556	-4.4863	-4.6301	-4.6995	-4.1978	0	0	0	0
M00096_:PBX-1	1.7375	1.6354	1.5552	1.6067	1.0699	0.64381	0.55087	0.47596	0.52427
M00097_:PAX-6	-8.0022	-7.7638	-8.1891	-8.2133	-7.6892	0	0	0	0
M00098_:PAX-2	-1.6714	-1.683	-1.7296	-1.6855	-1.4691	0	0	0	0
M00102_:CDP	-5.3952	-4.9058	-5.3392	-4.9407	-4.6663	0	0	0	0
M00104_:CDP:CR1	-1.1375	-1.0909	-1.1529	-1.1212	-0.12037	0	0	0	0
M00105_:CDP:CR3	-4.7524	-4.9692	-4.862	-5.3262	-4.8981	0	0	0	0
M00106_:CDP:CR3+HD	-0.31999	-0.36799	-0.35758	-0.40429	0.42629	-0.41995	-0.41995	-0.41995	-0.41995
M00109_:C/EBPBETA	0.14942	0.063477	0.024848	0.079046	-0.39955	0.14914	0.063456	0.024847	0.079005
M00113_:CREB	-0.84055	-0.8768	-0.8755	-0.81513	-0.26496	0	0	0	0
M00114_:TAX/CREB	-4.8017	-4.7796	-4.8731	-4.784	-4.2673	0	0	0	0
M00115_:TAX/CREB	-4.2419	-4.3344	-4.3093	-4.3856	-3.9647	0	0	0	0
M00116_:C/EBPALPHA	0.69173	0.63767	0.57371	0.6368	0.34472	0.34357	0.29087	0.22799	0.29002
M00117_:C/EBPBETA	0.0070977	-0.00084999	-0.0093756	-0.0062066	-0.16793	0.0070976	0	0	0
M00118_:C-MYC:MAX	-5.9717	-5.181	-5.2734	-5.4601	-4.5522	0	0	0	0
M00119_:MAX	-3.4086	-3.5986	-3.5557	-3.4492	-3.0112	0	0	0	0
M00121_:USF	-2.8013	-2.6614	-2.702	-2.5945	-2.4448	0	0	0	0
M00122_:USF	-0.56227	-0.55488	-0.49991	-0.5507	-0.65895	0	0	0	0
M00123_:C-MYC:MAX	-1.3175	-1.3664	-1.3142	-1.4082	-1.0825	0	0	0	0
M00124_:PBX1B	-5.5862	-4.8086	-4.4478	-4.7391	-5.477	0	0	0	0
M00126_:GATA-1	-0.36636	-0.40901	-0.43381	-0.39881	-0.28103	0	0	0	0
M00127_:GATA-1	0.22053	0.25577	0.21664	0.28285	0.28921	-0.068653	-0.033435	-0.07253	-0.0063526

M00128_:GATA-1	-0.30086	-0.35252	-0.31216	-0.30965	-0.36956	0	0	0	0
M00130_:FOXD3	0.15435	0.15627	0.12888	0.16902	-1.7953	0.15404	0.15596	0.12871	0.16862
M00132_:HNF-1	-3.3681	-3.7454	-3.8109	-3.7257	-4.4171	0	0	0	0
M00133_:TST-1	1.4343	1.4142	1.3418	1.4304	1.2932	0.14086	0.12083	0.048638	0.13704
M00134_:HNF-4	-4.1567	-3.9935	-3.8823	-4.1148	-4.4358	0	0	0	0
M00135_:OCT-1	-5.4476	-5.4137	-4.6942	-5.0136	-6.3808	0	0	0	0
M00136_:OCT-1	-1.9596	-2.2259	-2.3127	-2.2137	-2.556	0	0	0	0
M00137_:OCT-1	1.1636	1.0617	0.97085	1.0973	0.94939	0.21338	0.11223	0.021462	0.1476
M00138_:OCT-1	-3.5748	-3.6862	-3.8671	-3.6038	-5.491	0	0	0	0
M00143_:BSAP	-6.775	-6.627	-6.4483	-6.5908	-6.6721	0	0	0	0
M00144_:BSAP	-6.1509	-6.2275	-6.2736	-6.2752	-5.9781	0	0	0	0
M00145_:BRN-2	-0.67163	-0.82186	-0.85154	-0.79777	-1.0999	0	0	0	0
M00146_:HSF1	0.10697	0.020886	0.031091	0.015665	0.27116	-0.16382	-0.24898	-0.23892	-0.25411
M00147_:HSF2	1.2089	1.1621	1.1495	1.1595	1.4141	-0.20441	-0.25069	-0.26302	-0.25325
M00148_:SRY	3.4851	3.3888	3.3562	3.3676	2.8076	0.65268	0.56536	0.53521	0.54578
M00152_:SRF	-9.6737	-10.2008	-10.8185	-10.4571	-9.9641	0	0	0	0
M00155_:ARP-1	-2.6636	-2.4386	-2.5018	-2.452	-3.1328	0	0	0	0
M00156_:RORALPHA1	-2.1073	-2.0699	-2.0227	-2.0856	-2.2435	0	0	0	0
M00157_:RORALPHA2	-3.1924	-3.4166	-3.4086	-3.5942	-3.754	0	0	0	0
M00158_:COUP-TF:HNF-4	-2.9499	-2.8402	-2.9736	-2.9079	-3.1156	0	0	0	0
M00159_:C/EBP	1.6475	1.5903	1.5403	1.6502	1.2723	0.37086	0.3153	0.26637	0.37345
M00160_:SRY	0.53323	0.42382	0.30519	0.39066	-0.55157	0.52095	0.41759	0.30284	0.38577
M00161_:OCT-1	-1.5084	-1.5192	-1.6162	-1.5185	-1.953	0	0	0	0
M00162_:OCT-1	1.4994	1.4509	1.3807	1.4834	1.0784	0.41485	0.36822	0.30004	0.39955
M00172_:AP-1	0.62017	0.62442	0.61257	0.60354	0.60396	0.016208	0.020453	0.008604	-0.0004251
M00173_:AP-1	0.55137	0.54991	0.53342	0.51896	0.49543	0.055919	0.054463	0.037981	0.023521
M00174_:AP-1	0.18769	0.23912	0.26691	0.18119	0.15827	0.02942	0.080803	0.10853	0.022918
M00175_:AP-4	1.9527	2.0228	2.0497	2.0204	1.8799	0.072768	0.14264	0.16935	0.14025
M00176_:AP-4	1.98	2.0493	2.0753	2.043	1.9634	0.016651	0.08589	0.11182	0.079561
M00177_:CREB	-1.642	-1.6199	-1.6363	-1.6107	-1.0866	0	0	0	0
M00178_:CREB	-1.6775	-1.6558	-1.6242	-1.6211	-1.0856	0	0	0	0
M00179_:CRE-BP1	-0.85562	-0.81231	-0.81186	-0.78943	-0.38496	0	0	0	0
M00183_:C-MYB	1.8312	1.8331	1.8261	1.8048	2.0993	-0.26652	-0.26469	-0.27151	-0.29239
M00184_:MYOD	1.1652	1.2391	1.352	1.2406	0.94003	0.22421	0.29689	0.40625	0.29831
M00185_:NF-Y	-0.71757	-0.74009	-0.89313	-0.86392	-0.69064	0	0	0	0
M00186_:SRF	-3.7291	-3.5814	-3.6814	-3.5886	-3.2927	0	0	0	0

M00187_:USF	0.57196	0.62405	0.63147	0.61882	0.80689	-0.23386	-0.18233	-0.17498	-0.18752
M00188_:AP-1	-0.035222	-0.01269	-0.034095	-0.092647	-0.14908	0	0	0	0
M00189_:AP-2	-0.3653	-0.12616	-0.14076	-0.19152	-0.14204	0	0	0	0
M00190_:C/EBP	0.18863	0.11638	0.030927	0.14083	-0.12241	0.18808	0.11625	0.030924	0.14059
M00191_:ER	-3.9604	-3.9272	-3.9748	-3.9275	-4.2313	0	0	0	0
M00192_:GR	-2.5245	-2.5178	-2.4641	-2.4747	-2.8016	0	0	0	0
M00193_:NF-1	-2.1973	-2.12	-2.085	-2.1107	-2.3324	0	0	0	0
M00194_:NF-KAPPAB	-2.0231	-1.8518	-1.8221	-1.9182	-1.9702	0	0	0	0
M00195_:OCT-1	-1.9519	-2.065	-2.0216	-1.9491	-2.6127	0	0	0	0
M00196_:SP1	-1.5576	-1.4318	-1.6812	-1.6055	-3.1259	0	0	0	0
M00199_:AP-1	1.0753	1.1001	1.0919	1.0984	0.85874	0.21568	0.24017	0.23212	0.23853
M00201_:C/EBP	-2.3401	-2.3307	-2.4081	-2.322	-2.341	0	0	0	0
M00203_:GATA-X	1.0934	0.97846	0.97919	0.98803	0.85884	0.23346	0.11948	0.12021	0.12901
M00205_:GR	-3.109	-3.0463	-3.1079	-3.0917	-3.3625	0	0	0	0
M00206_:HNF-1	-3.058	-3.4157	-3.5635	-3.4079	-4.0123	0	0	0	0
M00208_:NF-KAPPAB	-3.0698	-3.1979	-3.041	-3.3527	-3.4173	0	0	0	0
M00209_:NF-Y	-2.2456	-2.1485	-2.1928	-2.165	-2.1801	0	0	0	0
M00210_:OCT-X	-2.153	-2.1504	-2.3185	-2.1799	-2.741	0	0	0	0
M00215_:SRF	-3.8218	-3.7921	-3.8159	-3.7117	-3.6785	0	0	0	0
M00216_:TATA	0.66812	0.59575	0.50442	0.61411	-0.090928	0.64433	0.57874	0.49399	0.59551
M00217_:USF	1.9378	1.9657	2.0059	1.9685	2.0981	-0.15998	-0.13221	-0.092079	-0.12942
M00220_:SREBP-1	-1.5735	-1.5067	-1.4873	-1.6097	-2.2288	0	0	0	0
M00221_:SREBP-1	-1.3798	-1.2895	-1.2608	-1.3965	-1.5469	0	0	0	0
M00222_:HAND1:E47	-2.5442	-2.573	-2.5885	-2.5152	-2.9112	0	0	0	0
M00223_:STATX	0.23211	0.1871	0.10563	0.16928	0.15268	0.079385	0.03442	-0.047034	0.016601
M00224_:STAT1	-10.5803	-9.8702	-10.7791	-10.7703	-9.2715	0	0	0	0
M00225_:STAT3	-12.4428	-10.5911	-12.0104	-12.7234	-11.0713	0	0	0	0
M00231_:MEF-2	-6.6426	-6.7972	-7.0102	-6.6854	-7.7252	0	0	0	0
M00232_:MEF-2	-6.9634	-6.6289	-6.7636	-6.5937	-7.7796	0	0	0	0
M00233_:MEF-2	-13.9069	-12.9806	-13.9619	-13.0303	-15.0216	0	0	0	0
M00235_:AHR:ARNT	-4.5757	-4.3077	-4.3821	-4.5571	-3.6448	0	0	0	0
M00236_:ARNT	-3.3361	-3.5795	-3.8344	-3.5568	-3.2954	0	0	0	0
M00237_:AHR:ARNT	-12.031	-10.6497	-11.6671	-11.3824	-9.0539	0	0	0	0
M00243_:EGR-1	-6.5192	-5.2696	-5.865	-5.6615	-4.7571	0	0	0	0
M00245_:EGR-3	-6.3077	-5.6673	-4.7744	-4.5724	-4.5916	0	0	0	0
M00246_:EGR-2	-6.0662	-5.1058	-4.9372	-4.8897	-4.3485	0	0	0	0

M00248_:OCT-1	-3.0086	-2.8504	-3.2116	-2.6369	-3.9095	0	0	0	0
M00249_:CHOP:C/EBPALPHA	-2.571	-2.6667	-2.8465	-2.7457	-2.6338	0	0	0	0
M00251_:XBP-1	-5.6755	-5.6453	-5.7428	-5.4705	-4.8989	0	0	0	0
M00252_:TATA	-1.7305	-1.8104	-1.8215	-1.7356	-1.9788	0	0	0	0
M00256_:NRSF	-15.1901	-10.4795	-13.4244	-14.6704	-14.3446	0	0	0	0
M00257_:RREB-1	-3.2208	-3.0116	-2.8221	-2.8353	-3.9639	0	0	0	0
M00258_:ISRE	-3.9668	-4.1335	-4.3007	-4.4636	-5.2694	0	0	0	0
M00260_:HLF	-0.52559	-0.66975	-0.75999	-0.66023	-0.46053	0	0	0	0
M00271_:AML-1A	2.1096	2.0944	2.0832	2.1153	1.9539	0.15544	0.14026	0.12917	0.16105
M00272_:P53	0.028983	0.21184	0.20912	0.18587	0.043703	-0.01472	0.16774	0.16504	0.14193
M00277_:LMO2:COMPLEX	-0.2699	-0.19514	-0.040061	-0.12147	-0.71729	0	0	0	0
M00278_:LMO2:COMPLEX	0.62684	0.58762	0.52457	0.58556	1.1698	-0.53002	-0.56629	-0.62375	-0.56819
M00279_:MIF-1	-3.6965	-3.7621	-3.875	-3.7473	-3.5006	0	0	0	0
M00280_:RFX1	-3.8709	-3.8514	-3.8009	-3.8402	-3.4266	0	0	0	0
M00281_:RFX1	-4.7244	-4.3629	-4.2124	-4.7068	-4.3263	0	0	0	0
M00284_:TCF11:MAFG	-6.4823	-6.8592	-6.7012	-6.9541	-7.2888	0	0	0	0
M00285_:TCF11	1.1967	1.1837	1.1155	1.1765	1.2452	-0.04845	-0.061469	-0.12947	-0.068619
M00287_:NF-Y	-2.8528	-2.8263	-3.2877	-3.4688	-3.4603	0	0	0	0
M00289_:HFH-3	-0.81016	-0.78615	-0.75254	-0.76173	-2.8287	0	0	0	0
M00290_:FREAC-2	-3.3287	-3.4277	-3.3679	-3.3873	-3.8076	0	0	0	0
M00291_:FREAC-3	-3.3088	-3.6826	-3.7776	-3.7246	-4.4215	0	0	0	0
M00292_:FREAC-4	-4.0696	-5.1711	-5.0113	-4.8903	-5.4632	0	0	0	0
M00293_:FREAC-7	-1.09	-1.0903	-1.3224	-1.0217	-3.0271	0	0	0	0
M00302_:NF-AT	0.70695	0.61422	0.6119	0.67074	-0.15853	0.67891	0.59561	0.59349	0.64667
M00322_:C-MYC:MAX	0.14711	0.27382	0.2536	0.21129	0.56493	-0.41185	-0.28908	-0.30883	-0.35
M00325_:NEURAL-RESTR.-SILENCER-ELEMENT	-8.5019	-8.1207	-7.6543	-8.2625	-7.9579	0	0	0	0
M00326_:PAX-1	-8.7015	-8.8243	-8.8759	-8.8478	-8.0834	0	0	0	0
M00327_:PAX-3	-5.1837	-5.1193	-5.2064	-5.1638	-4.2525	0	0	0	0
M00328_:PAX-8	-1.1872	-1.1982	-1.2204	-1.1745	-1.1614	0	0	0	0
M00332_:WHN	-1.7143	-1.751	-1.964	-1.8512	-0.44058	0	0	0	0
M00338_:ATF	-1.7393	-1.6541	-1.7263	-1.6059	-1.0311	0	0	0	0
M00339_:C-ETS-1	-1.658	-1.7986	-1.7155	-1.7928	-2.3038	0	0	0	0
M00340_:C-ETS-2	-0.74421	-0.85356	-0.75884	-0.7826	-1.1376	0	0	0	0
M00341_:GABP	-1.4873	-1.4437	-1.4805	-1.4223	-1.4056	0	0	0	0
M00342_:OCT-1	-1.0002	-1.125	-1.0637	-1.0501	-1.7304	0	0	0	0

M00346_:GATA-1	0.86313	0.75926	0.75108	0.75835	0.69264	0.17008	0.066597	0.058429	0.065691
M00403_:AMEF-2	-3.3111	-3.3138	-3.2983	-3.2815	-4.0918	0	0	0	0
M00406_:MEF-2	-5.2311	-5.1228	-4.8067	-4.993	-6.0251	0	0	0	0
M00407_:RSRFC4	-4.9469	-5.0317	-4.9923	-4.5794	-6.1795	0	0	0	0
M00410_:SOX-9	-1.609	-1.7393	-1.7535	-1.7917	-2.0938	0	0	0	0
M00412_:AREB6	-0.73961	-0.71956	-0.69191	-0.71039	-0.94143	0	0	0	0
M00413_:AREB6	0.0649	0.13195	0.18656	0.09904	-0.31756	0.064878	0.13176	0.18602	0.098959
M00414_:AREB6	-0.46628	-0.41121	-0.30191	-0.39252	-0.72944	0	0	0	0
M00415_:AREB6	2.4914	2.5248	2.452	2.5426	2.3925	0.09876	0.13202	0.059424	0.14977
M00416_:CART-1	-3.984	-4.0619	-4.3737	-3.8626	-4.7012	0	0	0	0
M00418_:TGIF	-0.42625	-0.32702	-0.39742	-0.33619	-0.44993	0	0	0	0
M00419_:MEIS1	0.065459	0.074046	0.065303	0.040464	-0.091743	0.065436	0.074012	0.06528	0.040458
M00420_:MEIS1A:HOXA9	-4.2553	-4.6482	-4.8044	-4.763	-4.6671	0	0	0	0
M00421_:MEIS1B:HOXA9	-3.9342	-3.9206	-4.0477	-4.0923	-4.196	0	0	0	0
M00422_:FOXJ2	-0.62844	-0.89193	-0.99375	-1.0036	-4.0377	0	0	0	0
M00423_:FOXJ2	-0.29648	-0.37243	-0.50436	-0.41198	-1.0675	0	0	0	0
M00424_:NKX6-1	-2.4405	-2.5646	-2.6229	-2.4968	-3.7565	0	0	0	0
M00425_:E2F	0.1842	0.31736	0.20663	0.26098	1.0194	-0.78979	-0.67453	-0.77077	-0.72402
M00426_:E2F	0.19699	0.31838	0.21524	0.27037	0.98793	-0.75214	-0.64561	-0.73641	-0.68828
M00427_:E2F	0.14159	0.26833	0.16118	0.20511	0.98171	-0.79396	-0.68459	-0.77739	-0.73979
M00428_:E2F-1	2.7018	2.7859	2.7627	2.7386	3.1599	-0.4502	-0.36966	-0.39199	-0.41511
M00431_:E2F-1	0.68982	0.78925	0.69973	0.71136	1.4484	-0.72417	-0.63627	-0.71555	-0.70538
M00432_:TTF1	0.98925	0.94534	0.88857	0.95781	0.81606	0.17276	0.1291	0.072477	0.14151
M00436_:IPF1	0.61719	0.56548	0.47744	0.57939	0.44189	0.17485	0.12344	0.035544	0.13728
M00437_:CHX10	-2.3364	-2.2392	-2.1845	-2.2712	-2.0883	0	0	0	0
M00444_:VDR	-0.52453	-0.60578	-0.4971	-0.6032	-1.2725	0	0	0	0
M00447_:AR	-2.3334	-2.4169	-2.4527	-2.3702	-2.6435	0	0	0	0
M00449_:ZIC2	1.315	1.3651	1.4037	1.3527	1.2276	0.087332	0.1373	0.17566	0.12501
M00451_:NKX3A	-0.56599	-0.64205	-0.80411	-0.61817	-1.4563	0	0	0	0
M00453_:IRF-7	-4.205	-4.3082	-4.3895	-4.3922	-4.7779	0	0	0	0
M00454_:MRF-2	-1.9768	-2.0262	-1.9147	-2.0925	-2.2157	0	0	0	0
M00456_:FAC1	0.60076	0.47582	0.4271	0.45092	-0.70155	0.58332	0.46704	0.42073	0.44344
M00457_:STAT5A:(HOMODIMER)	-2.3316	-2.5475	-2.5125	-2.468	-3.0124	0	0	0	0
M00459_:STAT5B:(HOMODIMER)	-3.9453	-4.0019	-4.0452	-4.0163	-4.7208	0	0	0	0
M00460_:STAT5A: (HOMOTETRAMER)	-8.3958	-7.8114	-8.1289	-8.2906	-8.4162	0	0	0	0

M00462_:GATA-6	2.3413	2.2628	2.2206	2.3032	2.4838	-0.1422	-0.22004	-0.26169	-0.18005
M00463_:POU3F2	-3.6853	-3.7182	-3.3299	-3.6874	-5.2499	0	0	0	0
M00464_:POU3F2	-0.3584	-0.44594	-0.57858	-0.37537	-0.87014	0	0	0	0
M00466_:HIF-1	-1.5873	-1.4901	-1.5262	-1.4836	-0.83383	0	0	0	0
M00468_:AP-2REP	3.4647	3.4887	3.491	3.4932	3.4754	-0.010688	0.01335	0.015678	0.017787
M00469_:AP-2ALPHA	1.8711	1.9787	2.0122	1.9605	1.7807	0.090387	0.1974	0.23052	0.17928
M00470_:AP-2GAMMA	1.2733	1.3496	1.3976	1.3477	1.0699	0.20265	0.27783	0.3248	0.27598
M00471_:TBP	1.4371	1.4313	1.2008	1.4964	0.55351	0.83023	0.82542	0.62556	0.8787
M00472_:FOXO4	0.8077	0.64509	0.5985	0.62136	-0.11609	0.76648	0.62361	0.58126	0.60211
M00473_:FOXO1	1.0593	0.92276	0.82182	0.87396	-0.11706	0.97021	0.86242	0.77849	0.82228
M00474_:FOXO1	-2.9844	-2.9758	-3.2011	-3.0402	-3.747	0	0	0	0
M00476_:FOXO4	-2.8495	-2.9999	-3.1503	-2.9804	-3.7439	0	0	0	0
M00477_:FOXO3	-2.8104	-2.7458	-2.9715	-2.705	-3.9305	0	0	0	0
M00478_:CDC5	-1.035	-1.1472	-1.2548	-1.1323	-1.4183	0	0	0	0
M00480_:LUN-1	-8.3866	-7.2964	-9.1913	-5.2197	-12.0928	0	0	0	0
M00481_:AR	-4.9852	-4.7006	-4.8662	-4.6711	-4.562	0	0	0	0
M00482_:PITX2	1.3296	1.3749	1.2974	1.2671	0.084952	1.1055	1.1365	1.0829	1.0614
M00483_:ATF6	-1.0925	-1.1315	-1.1017	-1.1167	-0.62092	0	0	0	0
M00484_:NCX	1.3769	1.3583	1.3109	1.3793	1.5064	-0.12926	-0.14783	-0.19487	-0.12688
M00485_:NKX2-2	-1.0013	-0.94849	-1.051	-0.98034	-1.3239	0	0	0	0
M00486_:PAX-2	3.3647	3.3189	3.2805	3.2811	3.0064	0.35459	0.31006	0.27238	0.27302
M00489_:NKX6-2	2.7649	2.6476	2.5576	2.658	2.1339	0.61088	0.50271	0.41749	0.51237
M00490_:BACH2	-1.5195	-1.4359	-1.6627	-1.431	-1.4992	0	0	0	0
M00491_:MAZR	-2.5002	-2.6161	-2.4463	-2.7631	-3.3671	0	0	0	0
M00492_:STAT1	1.2314	1.282	1.2789	1.238	1.3975	-0.16572	-0.11543	-0.11846	-0.15918
M00493_:STAT5A	3.2715	3.2508	3.1779	3.2739	2.9123	0.35534	0.33527	0.26403	0.35774
M00494_:STAT6	3.0276	3.009	2.9211	3.0307	2.7203	0.30483	0.28662	0.20008	0.30784
M00495_:BACH1	-4.7977	-4.4551	-4.6482	-4.3057	-4.7918	0	0	0	0
M00496_:STAT1	3.1609	3.1882	3.1155	3.2072	3.0386	0.12223	0.14936	0.076924	0.16828
M00497_:STAT3	3.472	3.4898	3.4655	3.4739	3.4161	0.055883	0.073703	0.049469	0.057839
M00498_:STAT4	3.0707	3.0602	2.9894	3.1038	2.7746	0.29402	0.28369	0.21399	0.32626
M00499_:STAT5A	3.6693	3.6805	3.6039	3.7138	3.2314	0.43102	0.44166	0.36819	0.47322
M00500_:STAT6	2.8758	2.9049	2.8759	2.9147	2.3888	0.47754	0.50494	0.47764	0.51408
M00510_:LHX3	-0.89099	-1.0691	-1.2388	-1.2002	-2.2352	0	0	0	0
M00511_:ERR:ALPHA	-1.4504	-1.4209	-1.4699	-1.4399	-1.7347	0	0	0	0
M00512_:PPARG	-12.686	-12.2565	-9.0784	-12.3016	-11.0092	0	0	0	0

M00513_:ATF3	-2.5	-2.4734	-2.414	-2.5143	-2.3124	0	0	0	0
M00514_:ATF4	0.29066	0.31611	0.33076	0.308	0.43224	-0.14134	-0.116	-0.10139	-0.12408
M00515_:PPARG	-14.7204	-13.9817	-15.9266	-14.6241	-14.9521	0	0	0	0
M00516_:E2F	-3.5108	-3.1452	-3.0978	-3.2523	-2.1146	0	0	0	0
M00517_:AP-1	-1.2313	-1.1772	-1.2135	-1.1735	-1.3111	0	0	0	0
M00518_:PPARALPHA:RXR-ALPHA	-6.0501	-6.4737	-6.1643	-6.4452	-6.7416	0	0	0	0
M00526_:GCNF	-9.134	-8.5746	-8.7288	-8.5469	-8.9834	0	0	0	0
M00528_:PPAR	-6.1517	-5.9734	-5.9091	-5.7877	-6.1686	0	0	0	0
M00531_:NERF1A	-2.3282	-2.3001	-2.1733	-2.2754	-2.5317	0	0	0	0
M00532_:RP58	-3.5011	-3.9234	-3.898	-3.4099	-4.0007	0	0	0	0
M00539_:ARNT	-5.1884	-4.9742	-5.121	-4.7017	-4.6903	0	0	0	0
M00615_:C-MYC:MAX	-3.4346	-3.4932	-3.4896	-3.4679	-2.9178	0	0	0	0
M00616_:AFP1	0.44544	0.35959	0.30752	0.35921	0.14035	0.30275	0.21837	0.16679	0.218
M00619_:ALX-4	-3.455	-3.5411	-3.572	-3.5722	-3.5416	0	0	0	0
M00621_:C/EBPDELTA	-0.15972	-0.20235	-0.25392	-0.1595	-0.44017	0	0	0	0
M00622_:C/EBPGAMMA	0.44596	0.38308	0.34187	0.42048	-0.26318	0.43872	0.37846	0.33858	0.4144
M00623_:CRX	-0.098845	-0.069466	-0.15423	-0.11554	-0.4569	0	0	0	0
M00624_:DBP	3.0041	2.9772	2.949	2.9809	2.8675	0.1364	0.10956	0.081484	0.11324
M00626_:RFX1:(EF-C)	-0.45394	-0.48638	-0.52205	-0.49608	-0.37526	0	0	0	0
M00631_:FXR/RXR-ALPHA	-2.1921	-2.1727	-2.1963	-2.207	-2.1219	0	0	0	0
M00632_:GATA-4	0.82577	0.7831	0.79108	0.78986	0.13939	0.66065	0.62237	0.62957	0.62846
M00634_:GCM	0.32471	0.37825	0.38826	0.36502	0.48228	-0.15724	-0.10393	-0.09395	-0.11712
M00638_:HNF-4ALPHA	-1.2797	-1.2827	-1.3481	-1.2743	-1.3508	0	0	0	0
M00639_:HNF-6	-0.77423	-0.86258	-1.0432	-0.94983	-1.7638	0	0	0	0
M00640_:HOXA4	3.3356	3.2808	3.2236	3.284	3.073	0.26116	0.20711	0.15037	0.21028
M00641_:HSF	-1.4437	-1.444	-1.4558	-1.4036	-1.515	0	0	0	0
M00644_:LBP-1	3.3758	3.4208	3.4537	3.4136	3.3381	0.037698	0.082729	0.11554	0.075548
M00646_:LF-A1	2.8762	2.9139	2.956	2.8962	2.7976	0.078487	0.11618	0.158	0.098476
M00647_:LXR	-5.4632	-5.3434	-5.4327	-5.4236	-5.5418	0	0	0	0
M00649_:MAZ	1.5017	1.4955	1.6764	1.5429	0.7348	0.73142	0.72606	0.87769	0.76678
M00650_:MTF-1	-3.1494	-3.1666	-3.1462	-3.2376	-3.3859	0	0	0	0
M00651_:NF-MUE1	-0.060744	0.043884	0.050045	0.087857	0.28872	-0.28674	-0.24362	-0.23755	-0.2002
M00652_:NRF-1	-1.0713	-0.87917	-0.96816	-1.0871	-0.39195	0	0	0	0
M00655_:PEA3	2.5714	2.5963	2.5908	2.5956	2.5914	-0.019997	0.0049573	-0.0005725	0.0042861
M00658_:PU.1	1.6654	1.6525	1.8209	1.6959	1.1733	0.48238	0.47021	0.62584	0.51102
M00665_:SP3	-0.83337	-0.75634	-0.70035	-0.75574	-0.97601	0	0	0	0

M00671_:TCF-4	2.5383	2.5675	2.4964	2.5921	2.401	0.13701	0.16605	0.095241	0.19051
M00672_:TEF	0.99981	0.92349	0.83375	0.93573	0.3251	0.65022	0.58115	0.49796	0.59234
M00678_:TEL-2	0.61769	0.64999	0.62015	0.63779	0.4565	0.16084	0.19288	0.16329	0.18079
M00687_:ALPHA-CP1	-1.6302	-1.6038	-1.6261	-1.6946	-1.6995	0	0	0	0
M00690_:AP-3	2.8222	2.8073	2.7767	2.8025	2.6039	0.21745	0.20278	0.1724	0.19803
M00691_:ATF-1	-1.1351	-1.033	-1.0699	-1.0393	-0.83004	0	0	0	0
M00693_:E12	0.19298	0.31405	0.40835	0.35235	0.042862	0.14984	0.26954	0.36147	0.30705
M00694_:E4F1	0.66045	0.69338	0.67897	0.67424	1.0472	-0.38199	-0.35017	-0.36412	-0.36869
M00695_:ETF	2.7487	2.8426	2.8653	2.8029	2.535	0.21285	0.30519	0.32731	0.26632
M00698_:HEB	2.2673	2.3163	2.3622	2.3064	2.1762	0.091021	0.13985	0.18546	0.13008
M00699_:ICSBP	-0.61706	-0.82158	-0.88161	-0.91815	-1.4728	0	0	0	0
M00701_:SMAD-3	0.73587	0.87182	0.95933	0.87749	0.61907	0.11666	0.25141	0.33701	0.25699
M00704_:TEF-1	4.2452	4.2364	4.2459	4.2576	4.1354	0.10974	0.10096	0.11046	0.12211
M00706_:TFII-I	2.6081	2.6063	2.7134	2.6257	2.2502	0.35411	0.35236	0.45507	0.37118
M00707_:TFIIA	1.5888	1.5927	1.5984	1.5952	1.6882	-0.099359	-0.095473	-0.0898	-0.093007
M00717_:PAX-8	0.30666	0.30976	0.27989	0.3314	0.35904	-0.052369	-0.049272	-0.07911	-0.027637
M00721_:CACCC-BINDING:FACTOR	-2.16	-2.1514	-2.1224	-2.2008	-2.4789	0	0	0	0
M00724_:HNF-3ALPHA	0.72954	0.74544	0.70446	0.81276	-0.71639	0.69881	0.71274	0.67671	0.77079
M00726_:USF2	4.0106	4.0675	4.0769	4.0623	4.1519	-0.14104	-0.084382	-0.07491	-0.089543
M00727_:SF-1	1.2973	1.3596	1.3105	1.3412	1.198	0.099221	0.16122	0.11236	0.14296
M00731_:OSF2	1.5955	1.628	1.636	1.6076	1.5068	0.088639	0.12103	0.12902	0.10074
M00733_:SMAD-4	-1.8467	-1.7042	-1.598	-1.7269	-1.8166	0	0	0	0
M00736_:E2F-1:DP-1	0.13279	0.19678	0.080966	0.078793	0.78274	-0.628	-0.56976	-0.67432	-0.67625
M00737_:E2F-1:DP-2	-0.4452	-0.33525	-0.46255	-0.45774	0.38328	-0.37866	-0.37866	-0.37866	-0.37866
M00738_:E2F-4:DP-1	-2.0503	-1.6897	-1.9471	-1.9722	-0.40749	0	0	0	0
M00739_:E2F-4:DP-2	-0.49736	-0.39759	-0.55607	-0.56879	0.49696	-0.48698	-0.48698	-0.48698	-0.48698
M00740_:RB:E2F-1:DP-1	-2.0281	-1.6538	-1.9323	-1.924	-0.28097	0	0	0	0
M00742_:HFH-4	-2.8187	-3.244	-3.3184	-2.9973	-5.409	0	0	0	0
M00744_:POU1F1	0.52766	0.38288	0.20866	0.40153	-0.43158	0.51575	0.37827	0.20791	0.39622
M00745_:LEF-1	0.29492	0.32535	0.30288	0.32803	0.40606	-0.11102	-0.080671	-0.10309	-0.077992
M00746_:ELF-1	0.37993	0.34968	0.36587	0.37468	-0.24924	0.37542	0.34616	0.36184	0.37035
M00747_:IRF1	2.319	2.3541	2.2532	2.4082	1.7028	0.59742	0.62919	0.53693	0.67752
M00750_:HMG:IY	2.9002	2.8779	2.8422	2.8916	2.6088	0.28941	0.26755	0.23237	0.28096
M00751_:AML1	2.7538	2.7595	2.7493	2.7884	2.571	0.18238	0.18798	0.1779	0.2166
M00761_:P53:DECAMER	1.0088	1.1027	1.1031	1.0813	0.91182	0.09695	0.19029	0.19066	0.16906
M00762_:PPAR,:HNF-4,:COUP,:RAR	-2.4338	-2.3299	-2.1496	-2.3113	-2.6618	0	0	0	0

M00763_:PPAR:DIRECT:REPEAT:1	-2.1269	-2.0427	-1.988	-2.0878	-2.2922	0	0	0	0
M00764_:HNF-4:DIRECT:REPEAT:1	-2.0005	-1.9504	-1.992	-1.9919	-2.1684	0	0	0	0
M00765_:COUP:DIRECT:REPEAT:1	-3.2594	-3.0588	-3.0784	-3.0719	-3.2245	0	0	0	0
M00766_:LXR:DIRECT:REPEAT:4	-4.188	-3.7826	-3.8491	-3.9148	-4.5203	0	0	0	0
M00767_:FXR:INVERTED:REPEAT:1	-2.1779	-2.1221	-2.192	-2.1995	-2.083	0	0	0	0
M00769_:AML	-2.0231	-1.9088	-1.8691	-1.812	-2.0584	0	0	0	0
M00770_:C/EBP	1.6868	1.6378	1.6036	1.6666	1.2932	0.38861	0.34125	0.30797	0.36911
M00771_:ETS	-1.1117	-1.0129	-0.95516	-0.9568	-1.5182	0	0	0	0
M00772_:IRF	-1.5805	-1.7663	-1.9473	-1.778	-3.3619	0	0	0	0
M00773_:MYB	1.4169	1.4657	1.4336	1.4378	1.7735	-0.35291	-0.30537	-0.33667	-0.33263
M00774_:NF-KAPPAB	-2.8171	-2.8236	-2.7748	-2.798	-3.1388	0	0	0	0
M00775_:NF-Y	-1.3078	-1.3127	-1.5179	-1.5913	-1.3377	0	0	0	0
M00776_:SREBP	-0.02405	0.03369	0.086527	-0.032689	-0.15079	0	0.033687	0.086473	0
M00777_:STAT	-0.13165	-0.14973	-0.19873	-0.13386	-0.59365	0	0	0	0
M00778_:AHR	-0.58626	-0.47693	-0.52799	-0.46388	0.069529	-0.069501	-0.069501	-0.069501	-0.069501
M00789_:GATA	2.019	1.9225	1.8939	1.948	1.9829	0.036065	-0.060357	-0.08891	-0.034866
M00790_:HNF-1	-3.7811	-3.9977	-3.9635	-3.8918	-4.9283	0	0	0	0
M00791_:HNF-3	0.80285	0.60026	0.52956	0.55175	-1.0922	0.76234	0.58286	0.51752	0.53817
M00792_:SMAD	3.1878	3.2156	3.213	3.1993	3.1718	0.015991	0.043738	0.041122	0.027493
M00793_:YY1	0.93049	1.0115	0.96504	1.013	0.66301	0.2659	0.34503	0.29975	0.34647
M00794_:TTF-1	1.1827	1.1797	1.1846	1.1626	0.89715	0.28366	0.28071	0.28546	0.26393
M00795_:OCTAMER	-0.35537	-0.41078	-0.56132	-0.37518	-1.2139	0	0	0	0
M00796_:USF	-1.4995	-1.3118	-1.2749	-1.3159	-1.3115	0	0	0	0
M00797_:HIF-1	-3.5077	-3.3089	-3.3267	-3.3426	-2.5042	0	0	0	0
M00799_:MYC	1.2939	1.3357	1.3914	1.359	1.6309	-0.33376	-0.29306	-0.23834	-0.27021
M00800_:AP-2	-1.8712	-1.6354	-1.6397	-1.6052	-1.9627	0	0	0	0
M00801_:CREB	1.9202	1.9906	1.9875	1.9489	2.621	-0.67339	-0.61025	-0.61314	-0.64783
M00802_:PIT-1	-3.066	-3.2466	-3.3883	-3.1655	-4.3437	0	0	0	0
M00803_:E2F	2.6042	2.7833	2.6477	2.6914	3.0452	-0.43403	-0.26043	-0.3924	-0.35019
M00804_:E2A	-0.19128	-0.15627	0.060658	-0.1574	-0.75804	0	0	0.060639	0
M00805_:LEF1	2.8228	2.8212	2.7819	2.8004	2.6039	0.21809	0.21648	0.17751	0.19585
M00806_:NF-1	-2.001	-1.8862	-1.9319	-1.919	-2.0265	0	0	0	0
M00807_:EGR	0.64434	0.72559	0.77278	0.71077	0.40003	0.24311	0.32272	0.36849	0.30827
M00808_:PAX	-1.8518	-1.6665	-1.6331	-1.612	-1.962	0	0	0	0
M00809_:FOX	-0.41978	-0.41504	-0.47908	-0.36606	-2.1035	0	0	0	0
M00810_:SRF	-5.6308	-5.5271	-5.5769	-5.2373	-5.4372	0	0	0	0

M00821_:NRF2	-1.4072	-1.3403	-1.3463	-1.3495	-1.6226	0	0	0	0
M00912_:C_EBP	0.57487	0.54979	0.49606	0.55259	0.16151	0.40758	0.38347	0.33147	0.38617
M00913_:MYB	2.3487	2.3466	2.3476	2.323	2.6553	-0.30423	-0.30625	-0.30532	-0.32922
M00915_:AP-2	-0.46628	-0.20954	-0.21441	-0.32927	-0.77677	0	0	0	0
M00916_:CREB	-2.4789	-2.4128	-2.3371	-2.327	-1.8264	0	0	0	0
M00917_:CREB	-1.413	-1.3931	-1.3461	-1.4044	-0.85834	0	0	0	0
M00918_:E2F	-1.1186	-0.92252	-1.0515	-1.0641	-0.061966	0	0	0	0
M00919_:E2F	-1.9165	-1.6313	-1.8337	-1.8153	-0.81326	0	0	0	0
M00920_:E2F	-2.778	-2.4578	-2.6161	-2.6405	-1.5154	0	0	0	0
M00921_:GR	2.2119	2.1954	2.211	2.2373	1.907	0.30258	0.28636	0.3017	0.32733
M00922_:SRF	-4.3266	-4.2916	-4.4291	-4.1193	-4.2113	0	0	0	0
M00924_:AP-1	0.28473	0.29549	0.33166	0.30498	0.26844	0.01629	0.027051	0.063202	0.036537
M00925_:AP-1	0.93861	0.93758	0.96976	0.93813	0.83533	0.10319	0.10216	0.13423	0.10271
M00926_:AP-1	1.1025	1.1284	1.0411	1.1496	0.98712	0.11523	0.14106	0.053981	0.16211
M00927_:AP-4	2.3685	2.4082	2.4575	2.4055	2.2987	0.069721	0.10933	0.15841	0.10663
M00929_:MYOD	-2.9935	-2.8589	-2.667	-2.7746	-3.357	0	0	0	0
M00930_:OCT-1	-0.23995	-0.28779	-0.43306	-0.24828	-1.2017	0	0	0	0
M00931_:SP-1	-0.42741	-0.45928	-0.5576	-0.47663	-1.8375	0	0	0	0
M00932_:SP-1	-1.4314	-1.3915	-1.5074	-1.5246	-2.9093	0	0	0	0
M00933_:SP-1	-0.34917	-0.043509	-0.021872	-0.13879	-1.5246	0	0	0	0
M00935_:NF-AT	0.87765	0.90919	0.93111	0.96579	0.44315	0.42779	0.45778	0.4785	0.51106
M00938_:E2F-1	-2.9751	-2.7901	-2.9306	-2.8336	-2.3752	0	0	0	0
M00939_:E2F-1	-1.4347	-1.2134	-1.355	-1.3939	-0.27929	0	0	0	0
M00940_:E2F-1	-0.85971	-0.71099	-0.852	-0.82765	0.060461	-0.060442	-0.060442	-0.060442	-0.060442
M00941_:MEF-2	-0.5311	-0.5484	-0.71116	-0.43324	-1.5021	0	0	0	0
M00947_:CP2/LBP-1C/LSF	-2.1547	-2.1073	-2.0727	-2.1311	-2.5455	0	0	0	0
M00954_:PR	-8.8296	-9.0211	-9.0019	-8.9376	-9.1844	0	0	0	0
M00955_:GR	-7.2558	-7.2152	-7.1417	-7.3986	-7.375	0	0	0	0
M00957_:PR	-8.7747	-8.8473	-8.6805	-8.8824	-8.952	0	0	0	0
M00959_:ER	-0.63432	-0.61338	-0.57502	-0.60284	-0.70506	0	0	0	0
M00960_:PR,:GR	1.4342	1.3727	1.3572	1.3907	1.0858	0.34496	0.28497	0.26976	0.30256
M00961_:VDR	0.77232	0.84606	0.85068	0.79925	0.86724	-0.094848	-0.02117	-0.016555	-0.067958
M00962_:AR	2.5615	2.5431	2.5539	2.5571	2.2684	0.29102	0.27298	0.28358	0.28672
M00963_:T3R	1.8435	1.9092	1.9184	1.8698	1.5066	0.33372	0.3973	0.40606	0.35928
M00964_:PXR,:CAR,:LXR,:FXR	0.088349	0.095049	0.046072	0.074666	-0.10317	0.088291	0.094977	0.046063	0.074632
M00965_:LXR,:PXR,:CAR,:COUP,:RA	-4.3582	-4.0357	-4.0297	-4.1111	-4.2765	0	0	0	0

R

M00966_:VDR,:CAR,:PXR	-5.5442	-5.5792	-5.5993	-5.6879	-5.7351	0	0	0	0
M00967_:HNF4,:COUP	1.4967	1.4863	1.4762	1.4456	1.3417	0.15471	0.1444	0.1343	0.10386
M00971_:ETS	1.3399	1.3992	1.402	1.3771	0.9516	0.38347	0.44023	0.44299	0.41916
M00972_:IRF	0.073194	0.06489	0.031737	0.10846	-1.4745	0.073162	0.064867	0.031735	0.10835
M00973_:E2A	1.5203	1.6076	1.7448	1.6187	1.1487	0.36734	0.451	0.57908	0.46156
M00974_:SMAD	0.53542	0.56794	0.60894	0.6077	0.27904	0.25499	0.2869	0.32694	0.32573
M00975_:RFX	2.195	2.2358	2.2306	2.2492	2.4258	-0.22979	-0.18947	-0.19462	-0.17618
M00976_:AHRHIF	0.36676	0.4731	0.43118	0.42623	1.0426	-0.65128	-0.55463	-0.5931	-0.59761
M00978_:LEF1TCF1	-0.35489	-0.29953	-0.45948	-0.27001	-0.59047	0	0	0	0
M00979_:PAX6	-5.824	-5.1418	-5.5484	-5.6043	-5.9943	0	0	0	0
M00980_:TBP	2.9287	2.9092	2.784	2.9415	2.2929	0.61522	0.59747	0.48139	0.62678
M00981_:CREBATF	-0.69639	-0.72078	-0.7777	-0.65802	0.078847	-0.078807	-0.078807	-0.078807	-0.078807
M00982_:KROX	-2.353	-2.541	-2.4691	-2.7057	-4.1998	0	0	0	0
M00983_:MAF	-0.22636	-0.22616	-0.22039	-0.24748	-0.75711	0	0	0	0
M00984_:PEBP	-2.3361	-2.2706	-2.2987	-2.2288	-2.2483	0	0	0	0
M00991_:CDX	-1.268	-1.5581	-1.6357	-1.6559	-2.8195	0	0	0	0
M00992_:FOXP3	-1.8475	-1.8951	-1.9323	-1.8749	-2.2105	0	0	0	0
M00993_:TAL1	1.9196	1.9375	2.003	1.9358	1.7691	0.15031	0.16806	0.23287	0.16632
M00997_:DEC	0.48329	0.54812	0.54162	0.53188	0.52579	-0.042489	0.022326	0.015828	0.0060945
M00998_:PBX	-0.036694	-0.0037915	0.044985	0.06485	0.039055	-0.03905	-0.03905	0.0059295	0.025793
M00999_:AIRE	-6.5218	-6.7255	-6.8829	-6.5168	-7.5139	0	0	0	0
M01000_:AIRE	-18.419	-17.6506	-17.541	-17.6677	-18.0916	0	0	0	0
M01007_:SRF	-6.3162	-6.5014	-6.4464	-6.3903	-6.5659	0	0	0	0
M01008_:EBOX	0.77818	0.91536	1.0093	0.92447	0.59177	0.18587	0.3208	0.4116	0.32967
M01009_:HES1	-0.12672	-0.043393	-0.034545	-0.075557	0.024184	-0.024183	-0.024183	-0.024183	-0.024183
M01010_:HMGIY	0.65528	0.65503	0.55299	0.71084	-0.5902	0.6328	0.63258	0.53932	0.68235
M01011_:HNF1	-4.3659	-4.4264	-4.5217	-4.4944	-5.7686	0	0	0	0
M01012_:HNF3	-1.1338	-1.159	-1.0911	-1.0654	-3.1116	0	0	0	0
M01013_:IPF1	-1.6851	-1.6002	-1.7271	-1.6377	-1.5314	0	0	0	0
M01014_:SOX	-0.7906	-0.80799	-0.87331	-0.80072	-1.1465	0	0	0	0
M01017_:PBX1	0.22786	0.25105	0.2244	0.19645	0.24822	-0.020361	0.0028359	-0.023814	-0.05176
