# Supplementary material for: Quantitative model for inferring dynamic regulation of the tumour suppressor gene p53
Source: BMC Bioinformatics. 2010 Jan 19;11:36. doi: 10.1186/1471-2105-11-36 (PMC2832896; doi:10.1186/1471-2105-11-36)
Supplement: Additional file 4 — Hierarchical clustering of relative sequence affinity ratios. Here we present results of hierarchical clustering of relative sequence affinity ratios for 409 human TFs across four list of putative p53 target genes. Yellow colour represents enriched TFs but blue colour represents depleted TFs. [file 1471-2105-11-36-S4.PDF]

Supplementary Figure 1.

Hierarchical clustering of relative sequence affinity ratios

Here we present results of hierarchical clustering of relative sequence affinity ratios for 409 human TFs across four list of putative p53 target genes.

Yellow colour represents enriched TFs but blue colour represents depleted TFs.

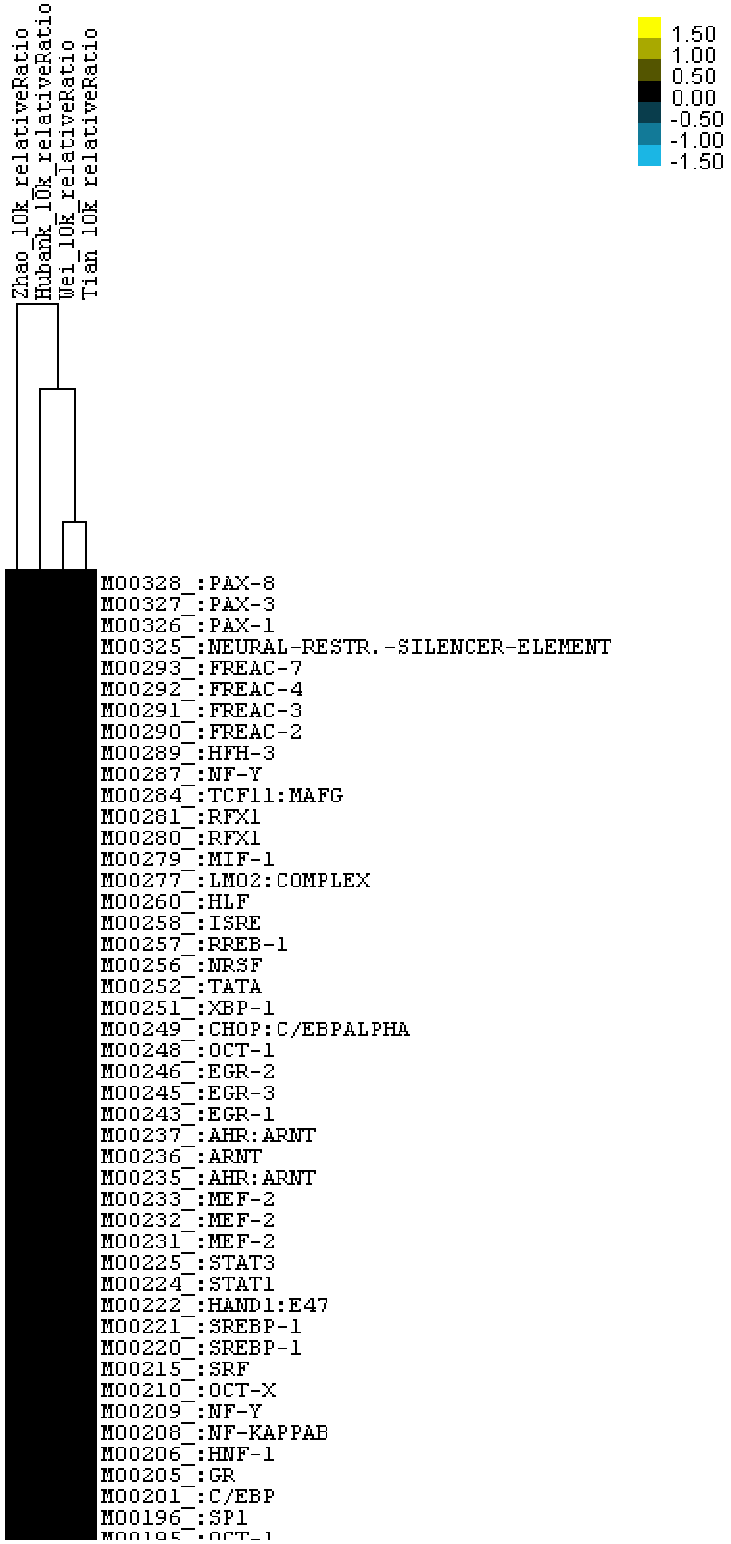

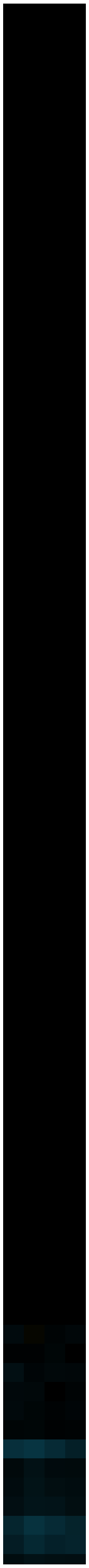

[illegible]
M00201_:C/EBP
M00196_:SP1
M00195_:OCT-1
M00194_:NF-KAPPAB
M00193_:NF-1
M00192_:GR
M00191_:ER
M00189_:AP-2
M00188_:AP-1
M00186_:SRF
M00185_:NF-Y
M00179_:CRE-BP1
M00178_:CREB
M00177_:CREB
M00161_:OCT-1
M00158_:COUP-TF:HNF-4
M00157_:RORALPHA2
M00156_:RORALPHA1
M00155_:ARP-1
M00152_:SRF
M00145_:BRN-2
M00144_:BSAP
M00143_:BSAP
M00138_:OCT-1
M00136_:OCT-1
M00135_:OCT-1
M00134_:HNF-4
M00132_:HNF-1
M00128_:GATA-1
M00126_:GATA-1
M00124_:PBX1B
M00123_:C-MYC:MAX
M00122_:USF
M00121_:USF
M00119_:MAX
M00118_:C-MYC:MAX
M00115_:TAX/CREB
M00114_:TAX/CREB
M00113_:CREB
M00105_:CDP:CR3
M00104_:CDP:CR1
M00102_:CDP
M00098_:PAX-2
M00097_:PAX-6
M00095_:CDP
M00085_:ZID
M00084_:MZF1
M00071_:E47
M00070_:TAL-1BETA:ITF-2
M00069_:YY1
M00066_:TAL-1ALPHA:E47
M00065_:TAL-1BETA:E47
M00062_:IRF-1
M00056_:MYOGENIN:/:NF-1
M00054_:NF-KAPPAB
M00052_:NF-KAPPAB:(P65)
M00051_:NF-KAPPAB:(P50)
M00045_:E4BP4
M00041_:CRE-BP1:C-JUN
M00040_:CRE-BP1
M00037_:NF-E2
M00034_:P53
M00026_:RSRFC4
M00025_:ELK-1
M00024_:E2F
M00017_:ATF
M00006_:MEF-2
M00005_:AP-4
M00002_:E47
M00789_:GATA
M01017_:PBX1
M00285_:TCF11
M00127_:GATA-1
M00717_:PAX-8
M00077_:GATA-3
M00803_:E2F
M00726_:USF2
M00634_:GCM
M00492_:STAT1
M00322_:C-MYC:MAX
M00799_:MYC
[illegible]

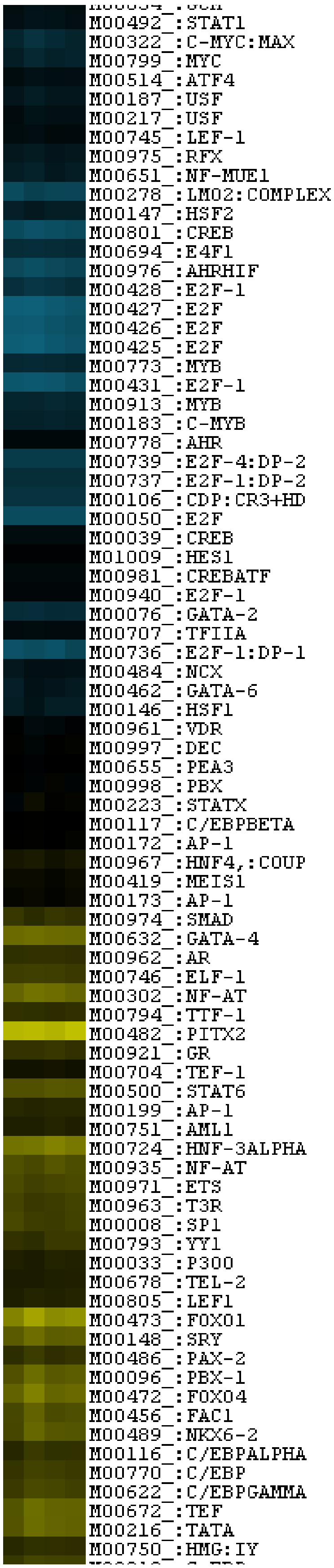
M00492_:STAT1
M00322_:C-MYC:MAX
M00799_:MYC
M00514_:ATF4
M00187_:USF
M00217_:USF
M00745_:LEF-1
M00975_:RFX
M00651_:NF-MUE1
M00278_:LMO2:COMPLEX
M00147_:HSF2
M00801_:CREB
M00694_:E4F1
M00976_:AHRHIF
M00428_:E2F-1
M00427_:E2F
M00426_:E2F
M00425_:E2F
M00773_:MYB
M00431_:E2F-1
M00913_:MYB
M00183_:C-MYB
M00778_:AHR
M00739_:E2F-4:DP-2
M00737_:E2F-1:DP-2
M00106_:CDP:CR3+HD
M00050_:E2F
M00039_:CREB
M01009_:HES1
M00981_:CREBATF
M00940_:E2F-1
M00076_:GATA-2
M00707_:TFIIA
M00736_:E2F-1:DP-1
M00484_:NCX
M00462_:GATA-6
M00146_:HSF1
M00961_:VDR
M00997_:DEC
M00655_:PEA3
M00998_:PBX
M00223_:STATX
M00117_:C/EBPBETA
M00172_:AP-1
M00967_:HNF4,:COUP
M00419_:MEIS1
M00173_:AP-1
M00974_:SMAD
M00632_:GATA-4
M00962_:AR
M00746_:ELF-1
M00302_:NF-AT
M00794_:TTF-1
M00482_:PITX2
M00921_:GR
M00704_:TEF-1
M00500_:STAT6
M00199_:AP-1
M00751_:AML1
M00724_:HNF-3ALPHA
M00935_:NF-AT
M00971_:ETS
M00963_:T3R
M00008_:SP1
M00793_:YY1
M00033_:P300
M00678_:TEL-2
M00805_:LEF1
M00473_:FOXO1
M00148_:SRY
M00486_:PAX-2
M00096_:PBX-1
M00472_:FOXO4
M00456_:FAC1
M00489_:NKX6-2
M00116_:C/EBPALPHA
M00770_:C/EBP
M00622_:C/EBPGAMMA
M00672_:TEF
M00216_:TATA
M00750_:HMG:IY

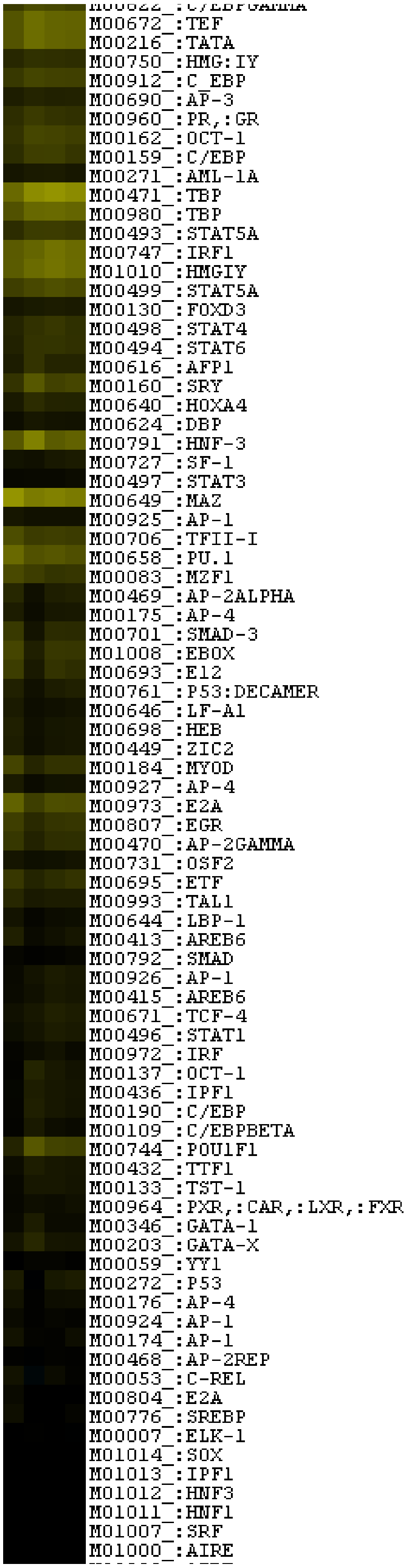
M00672_:TEF
M00216_:TATA
M00750_:HMG:IY
M00912_:C_EBP
M00690_:AP-3
M00960_:PR,:GR
M00162_:OCT-1
M00159_:C/EBP
M00271_:AML-1A
M00471_:TBP
M00980_:TBP
M00493_:STAT5A
M00747_:IRF1
M01010_:HMGIY
M00499_:STAT5A
M00130_:FOXD3
M00498_:STAT4
M00494_:STAT6
M00616_:AFP1
M00160_:SRY
M00640_:HOXA4
M00624_:DBP
M00791_:HNF-3
M00727_:SF-1
M00497_:STAT3
M00649_:MAZ
M00925_:AP-1
M00706_:TFII-I
M00658_:PU.1
M00083_:MZF1
M00469_:AP-2ALPHA
M00175_:AP-4
M00701_:SMAD-3
M01008_:EBOX
M00693_:E12
M00761_:P53:DECAMER
M00646_:LF-A1
M00698_:HEB
M00449_:ZIC2
M00184_:MYOD
M00927_:AP-4
M00973_:E2A
M00807_:EGR
M00470_:AP-2GAMMA
M00731_:OSF2
M00695_:ETF
M00993_:TAL1
M00644_:LBP-1
M00413_:AREB6
M00792_:SMAD
M00926_:AP-1
M00415_:AREB6
M00671_:TCF-4
M00496_:STAT1
M00972_:IRF
M00137_:OCT-1
M00436_:IPF1
M00190_:C/EBP
M00109_:C/EBPBETA
M00744_:POU1F1
M00432_:TTF1
M00133_:TST-1
M00964_:PXR,:CAR,:LXR,:FXR
M00346_:GATA-1
M00203_:GATA-X
M00059_:YY1
M00272_:P53
M00176_:AP-4
M00924_:AP-1
M00174_:AP-1
M00468_:AP-2REP
M00053_:C-REL
M00804_:E2A
M00776_:SREBP
M00007_:ELK-1
M01014_:SOX
M01013_:IPF1
M01012_:HNF3
M01011_:HNF1
M01007_:SRF
M01000_:AIRE

M01012_:HNF3
M01011_:HNF1
M01007_:SRF
M01000_:AIRE
M00999_:AIRE
M00992_:FOXP3
M00991_:CDX
M00984_:PEBP
M00983_:MAF
M00982_:KROX
M00979_:PAX6
M00978_:LEF1TCF1
M00966_:VDR,:CAR,:PXR
M00965_:LXR,:PXR,:CAR,:COUP,:RAR
M00959_:ER
M00957_:PR
M00955_:GR
M00954_:PR
M00947_:CP2/LBP-1C/LSF
M00941_:MEF-2
M00939_:E2F-1
M00938_:E2F-1
M00933_:SP-1
M00932_:SP-1
M00931_:SP-1
M00930_:OCT-1
M00929_:MYOD
M00922_:SRF
M00920_:E2F
M00919_:E2F
M00918_:E2F
M00917_:CREB
M00916_:CREB
M00915_:AP-2
M00821_:NRF2
M00810_:SRF
M00809_:FOX
M00808_:PAX
M00806_:NF-1
M00802_:PIT-1
M00800_:AP-2
M00797_:HIF-1
M00796_:USF
M00795_:OCTAMER
M00790_:HNF-1
M00777_:STAT
M00775_:NF-Y
M00774_:NF-KAPPAB
M00772_:IRF
M00771_:ETS
M00769_:AML
M00767_:FXR:INVERTED:REPEAT:1
M00766_:LXR:DIRECT:REPEAT:4
M00765_:COUP:DIRECT:REPEAT:1
M00764_:HNF-4:DIRECT:REPEAT:1
M00763_:PPAR:DIRECT:REPEAT:1
M00762_:PPAR,:HNF-4,:COUP,:RAR
M00742_:HFH-4
M00740_:RB:E2F-1:DP-1
M00738_:E2F-4:DP-1
M00733_:SMAD-4
M00721_:CACCC-BINDING:FACTOR
M00699_:ICSBP
M00691_:ATF-1
M00687_:ALPHA-CP1
M00665_:SP3
M00652_:NRF-1
M00650_:MTF-1
M00647_:LXR
M00641_:HSF
M00639_:HNF-6
M00638_:HNF-4ALPHA
M00631_:FXR/RXR-ALPHA
M00626_:RFX1:(EF-C)
M00623_:CRX
M00621_:C/EBPDELTA
M00619_:ALX-4
M00615_:C-MYC:MAX
M00539_:ARNT
M00532_:RP58
M00531_:NERF1A
M00528_:PPAR

M00539_:ARNT
M00532_:RP58
M00531_:NERF1A
M00528_:PPAR
M00526_:GCNF
M00518_:PPARALPHA:RXR-ALPHA
M00517_:AP-1
M00516_:E2F
M00515_:PPARG
M00513_:ATF3
M00512_:PPARG
M00511_:ERR:ALPHA
M00510_:LHX3
M00495_:BACH1
M00491_:MAZR
M00490_:BACH2
M00485_:NKX2-2
M00483_:ATF6
M00481_:AR
M00480_:LUN-1
M00478_:CDC5
M00477_:FOXO3
M00476_:FOXO4
M00474_:FOXO1
M00466_:HIF-1
M00464_:POU3F2
M00463_:POU3F2
M00460_:STAT5A:(HOMOTETRAMER)
M00459_:STAT5B:(HOMODIMER)
M00457_:STAT5A:(HOMODIMER)
M00454_:MRF-2
M00453_:IRF-7
M00451_:NKX3A
M00447_:AR
M00444_:VDR
M00437_:CHX10
M00424_:NKX6-1
M00423_:FOXJ2
M00422_:FOXJ2
M00421_:MEIS1B:HOXA9
M00420_:MEIS1A:HOXA9
M00418_:TGIF
M00416_:CART-1
M00414_:AREB6
M00412_:AREB6
M00410_:SOX-9
M00407_:RSRFC4
M00406_:MEF-2
M00403_:AMEF-2
M00342_:OCT-1
M00341_:GABP
M00340_:C-ETS-2
M00339_:C-ETS-1
M00338_:ATF
M00332_:WHN
